# The Diagnostic Performance of Coronary Artery Angiography with 64-MSCT and Post 64-MSCT: Systematic Review and Meta-Analysis

**DOI:** 10.1371/journal.pone.0084937

**Published:** 2014-01-21

**Authors:** Min Li, Xiang-min Du, Zhi-tao Jin, Zhao-hui Peng, Juan Ding, Li Li

**Affiliations:** 1 Department of Medical Imaging, Jinan Military General Hospital, Jinan, Shandong Province, China; 2 Department of Medical Engineering, Jinan Military General Hospital, Jinan, Shandong Province, China; 3 Department of Cardiology, General Hospital of the Second Artillery, Beijing, China; University of Groningen, Netherlands

## Abstract

**Purpose:**

To comprehensively investigate the diagnostic performance of coronary artery angiography with 64-MDCT and post 64-MDCT.

**Materials and Methods:**

PubMed was searched for all published studies that evaluated coronary arteries with 64-MDCT and post 64-MDCT. The clinical diagnostic role was evaluated by applying the likelihood ratios (LRs) to calculate the post-test probability based on Bayes' theorem.

**Results:**

91 studies that met our inclusion criteria were ultimately included in the analysis. The pooled positive and negative LRs at patient level were 8.91 (95% CI, 7.53, 10.54) and 0.02 (CI, 0.01, 0.03), respectively. For studies that did not claim that non-evaluable segments were included, the pooled positive and negative LRs were 11.16 (CI, 8.90, 14.00) and 0.01 (CI, 0.01, 0.03), respectively. For studies including uninterruptable results, the diagnostic performance decreased, with the pooled positive LR 7.40 (CI, 6.00, 9.13) and negative LR 0.02 (CI, 0.01, 0.03). The areas under the summary ROC curve were 0.98 (CI, 0.97 to 0.99) for 64-MDCT and 0.96 (CI, 0.94 to 0.98) for post 64-MDCT, respectively. For references explicitly stating that the non-assessable segments were included during analysis, a post-test probability of negative results >95% and a positive post-test probability <95% could be obtained for patients with a pre-test probability of <73% for coronary artery disease (CAD). On the other hand, when the pre-test probability of CAD was >73%, the diagnostic role was reversed, with a positive post-test probability of CAD >95% and a negative post-test probability of CAD <95%.

**Conclusion:**

The diagnostic performance of post 64-MDCT does not increase as compared with 64-MDCT. CTA, overall, is a test of exclusion for patients with a pre-test probability of CAD<73%, while for patients with a pre-test probability of CAD>73%, CTA is a test used to confirm the presence of CAD.

## Introduction

Coronary artery disease (CAD) is the leading illness threating human health in developed countries and it is increasingly becoming a significant public health problem in developing countries [1]. With the development of the 16-multi-detector CT (MDCT), a non-invasive approach of coronary CT angiography (CTA), it has been applied widely to avoid the complications of invasive coronary angiography (ICA), which is generally believed to be the gold standard in evaluating CAD [2].

Several meta-analysis studies have proven that single source 64-MDCT with improved parameters has a better ability to predict the stenosis of coronary artery lumen than that of 16-MDCT [3]–[5]. With the emergence and wider application of dual source 64-, 128-, 256-, and 320-MDCT it is hoped that the improvement will lead to a greater diagnostic accuracy than 64-MDCT. To our knowledge, no study has statistically proven that this claim is correct.

In particular, the CAD diagnosis is not only dependent upon the accuracy of CTA, but also upon pre-test probability, which is estimated according to the symptoms and examinations [3], [6]–[8]. The pre-test probability categorization is important because of its significant impact on the post-test probability of disease and the selection of a diagnostic test [8]. Appropriate application of CTA may improve patients' clinical outcomes, while the inappropriate utilization of CTA may generate extra radiation exposure to patients and unwarranted costs. Since there are already a number of risk algorithms available to evaluate the detailed pre-test probability [9]–[12], we evaluated the diagnostic role of CTA based on the diagnostic performance of CTA and the precise pre-test probability to provide a more practical patient-relevant utility of CTA.

## Materials and Methods

Generally, we followed a standard protocol based on the Preferred Reporting Items for Systematic Reviews and Meta-analyses (PRISMA) statement [13].

### Selection of Studies

PubMed was searched for all published studies that examined patients with 64-MDCT and post-64-MDCT. The language was limited to English and the search terms were, “computed tomography,” “multi-slice computed tomography,” “multi-section computed tomography,” “multi-detector computed tomography,” and “coronary angiography.”

The literature search ranged from 2004 to 2013, as 64-MDCT was first introduced into clinical practice in 2004. The references of published systematic reviews and meta-analyses were also screened. Two readers examined the studies to exclude potential duplicate or overlapping data.

### Study Eligibility

The title and abstract were reviewed first. If considered suitable or in doubt, the full text was screened. The inclusion criterion were listed as follows: 64-MDCT or post-64-MDCT was applied as a test to diagnose stenostic CAD (stenosis >50%); the absolute numbers of true-positive, false-positive, true-negative, and false-negative results were presented or can be calculated from the detailed data; and ICA served as the reference standard. Studies were excluded for the following reasons: they included patients who had undergone bypass graft surgery (CABG), percutaneous coronary intervention (PCI), or prior heart transplantation; they were retrospective studies.

### Data Extraction

Two investigators extracted the data independently. The following information was extracted from each study: first author, year of publication, country, number of patients, sex, age, heart rate, calcium scoring, the type and brand of machine used, temporal resolution, electrocardiographic (ECG) triggered scanning protocols, prevalence of CAD as well as non-diagnostic segments, and numbers of true-positive, true-negative, false-positive, and false-negative values. While most studies applied a 15-segment scheme of the coronary artery tree, several articles used other alternative protocols, such as 13-, 14-, 16-, and 17-segment models. The scheme of the coronary arterial tree for stenostic analysis was also extracted. Two readers assessed methodological quality independently and according to the QUADAS items [14].

### Data Synthesis and Statistical Analysis

Cohen κ test was conducted to evaluate the inter-observer agreement. The publication bias was assessed by the method developed by Deeks, et al. [15]. The heterogeneity across studies was evaluated by I^2^ test [16] and the impact of potential covariates was examined using meta-regression. Possible sources of heterogeneity were predefined based on QUADAS items, average age, gender, vendor, temporal resolution, number of slices, the scheme used to evaluate the coronary arterial tree, non-assessable segments, calcium score, protocol of ECG-triggered scanning, prevalence of CAD, and study quality score. A hierarchical summary receiver operating characteristic curves (sROC) was conducted based on the parameters estimated by the bivariate model. The area under the sROC (AUROC) serves as a global measure of CTA performance [17].

The available data was synthesized by an exact binomial rendition of the bivariate mixed-effects regression model [18]-[20]. We mainly calculated the positive and negative likelihood ratios (LRs) and 95% confidence intervals (CIs) to eliminate the influence of the prevalence of CAD [21] and to compute post-test probability. The LRs indicate by how much a given test would raise or lower the probability of having the disease.

Furthermore, the clinical, or patient-relevant, utility of the diagnostic test was evaluated by using the LRs and the pre-test probability of CAD to calculate the post-test probability based on Bayes' theorem [22]. The log-odds of the posterior probability, which show the chance to diagnose the disease after the test, is a linear function of the log-odds of prior probability and the log likelihood ratio of the target test, which is depicted visually with Fagan's nomogram [23]. The Fagan's nomogram plots an axis on the left with the prior log-odds, an axis in the middle representing the log likelihood ratio of the test, and an axis on the right representing the posterior log-odds. A straight line is drawn from the prior probability on the left axis through the likelihood ratios in the middle and extended to the posterior probability on the right. Thus, the posterior probability is estimated from the prior probability and the likelihood ratio of the test. The pre-test and post-test probabilities are both subjective estimates of the presence of a disease before and after a diagnostic test. The detailed pre-test probability of CAD could be calculated from clinical data and one or more proceeding tests [9], [10]. The post-test probability, in turn, can be calculated, depending on whether CTA falls out as a positive test or a negative test. If the positive or negative post-test probability is larger than 95%, the test is treated as an effective tool to confirm or exclude CAD. The overall diagnostic role of CTA at artery and segment levels was represented graphically by an LR scattergram introduced by Stengel et al. [24].

The data was analyzed using STATA (version 12), MetaDiSc (version 1.4), as well as SPSS (version 16.0).

## Results

### The Characteristics of CT Studies

**Figure 1:**
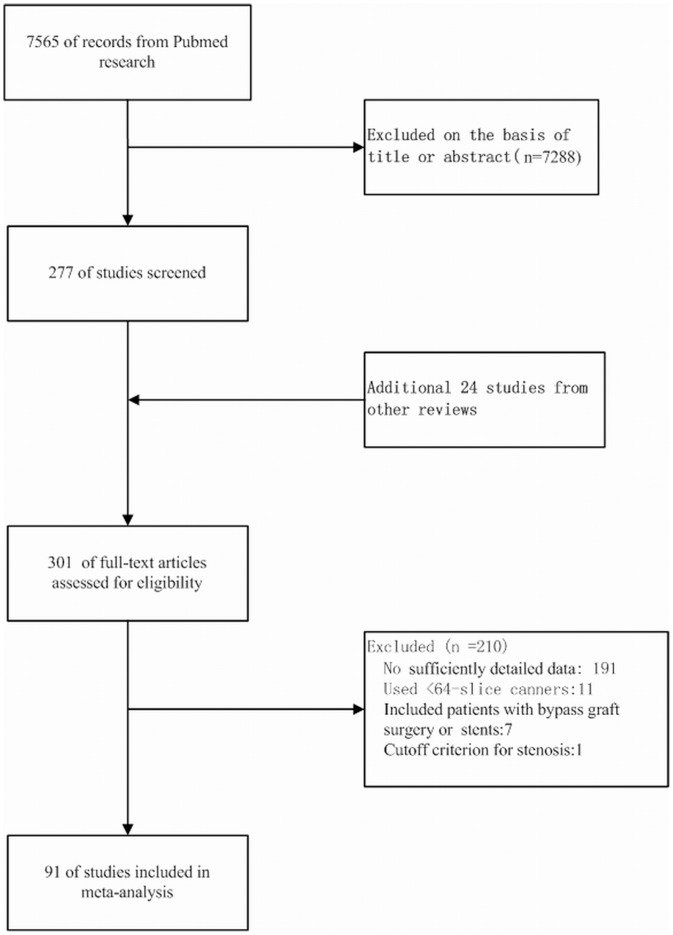
The flow chart for references searching and selection. After careful searching and selection, 91 studies were finally included in the analysis.

91 studies that met our inclusion criteria were finally included in the analysis (Figure 1 shows the literature search and selection algorithm). 48 studies performed CTA with single source, 64-MDCT; 26 studies with dual source, 64-MDCT; 6 with dual source, 128-MDCT; 9 with single source, 320-MDCT; 1 with single and dual source, 64-MDCT; and 1 with single source, 64-MDCT and 320-MDCT. 55 studies scanned the coronary artery with retrospective ECG gating, 21 studies with prospective protocol, 4 studies with retrospective and prospective ECG gating, and 11 studies did not report the detailed information. 36 studies reported a calcium score ranging from 47.7 to 821. The radiation dose ranged from 0.76 mSv to 21.4 mSv. Further information on the characteristics of each study is illustrated in Table 1.

**Table 1:** Characteristics of Included Studies.

Authors	Year	Country	Male/female	Mean age(y)	Scanner	Temporal resolusion(ms)	ECG triggered protocol	NO. of slice	HR(bpm)	Calcium score	Undiagnosis segments	Scheme to evaluate coronary tree	Radiationdose(mSv)
Leber[41]	2005	Germany	…	64	Siemens	165	R	64	62	…	0	15	10–14
Leschka[42]	2005	Switzerland	2.84	60	Siemens	165	R	64	66	…	0	15	…
Mollet[43]	2005	Netherlands	1.89	58	Siemens	165	R	64	58	231	0	16	15.2–21.4
Pugliese[44]	2005	Netherlands	1.5	61	Siemens	165	R	64	58		0.03	17	15–20
Raff[45]	2005	United States	3.12	59	Siemens	165	R	64	65	326	0.12	15	13–18
Ehara[46]	2006	Japan	3.06	67	Siemens	165	R	64	72	…	0.05	14	…
Ghostine[47]	2006	France	1.54	69	Siemens	165	R	64	67	…	0.06	15	7
Meijboom[48]	2006	Netherlands	2.33	63	Siemens	165	R	64	60	214	…	17	15.2 –21.4
Nikolaou[49]	2006	Germany	4.54	63	Siemens	165	R	64	61	…	0.1	15	8.8–13.6
Plass[50]	2006	Switzerland	3.54	66	Siemens	165	R	64	65	…	0.03	11	…
Ropers[51]	2006	Germany	1.63	58	Siemens	83	R	64	59	…	0.04	17	7.45–10.24
Scheffel[52]	2006	Switzerland	4	63.1	Siemens	83	R	64	70.3	821	0.014	…	…
Schuijf[53]	2006	Netherlands	3.29	60	Toshiba	200	R	64	60	423	0.014	…	…
Cademartiri[54]	2007	Italy	1.12	53.9	Siemens	165	R	64	60.6	47.7	0.004	17	15.2–21.4
Herzog[55]	2007	Germany	1.12	67	Siemens	165	R	64	64	…	0.076	15	…
Herzog[56]	2007	Germany	1.22	61	Siemens	165	R	64	72	…	…	15	…
Heuschmid[57]	2007	Germany	2.64	64	Siemens	83	R	64	65	779	0.046	13	…
Johnson[58]	2007	Germany	2.18	60	Siemens	83	R	64	68	…	0.02	15	4.6–7.8
Leber[59]	2007	Germany	1.73	58	Siemens	83	R	64		…	…	13	9.6
Meijboom[60]	2007	Netherlands	2.06	56.2	Siemens	165	R	64	59	…	…	17	13.4–17
Meijboom[61]	2007	Netherlands	2.59	58	Siemens	165	R	64	58	450	…	17	15.2–21.4
Oncel1[62]	2007	Turkey	3.21	56	Siemens	165	R	64	58	…	0	15	…
Ropers[63]	2007	Italy	1.70	61	Siemens	83	R	64	64	…	…	…	15.3
Scheffel[64]	2007	Switzerland	3.17	54	Siemens	165	…	64	65.5	136	0.018	15	8.8–13.6
Schlosser[65]	2007	Germany	1.86	62.4	Siemens	165	…	64	57	…	0.01	15	…
Weustink[66]	2007	Netherlands	3.76	61	Siemens	83	R	64	68	…	0.06	17	11.1–14.4
Achenbach[67]	2008	Germany	0.8	65	Siemens	165	R	64	64.5	…	0.12	17	12.6
Achenbach[67]	2008	Germany	1.78	61	Siemens	83	R	64	64.3	…	0.04	17	14.7
Alkadhi[68]	2008	Switzerland	2.19	62.9	Siemens	83	R	64	68.5	309	0.019	16	7–9
Brodoefel[69]	2008	Germany	4	62	Siemens	83	R	64	64.9	786.5	0.098	15	…
Han[70]	2008	Taiwan	…	59.64	GE	175	R	64	…	…	…	15	…
Herzog[71]	2008	Switzerland	1.73	58.8	GE	175	P	64	55.7	…	0.04	16	2.1
Leschka[72]	2008	Switzerland	0.54	64.1	Siemens	165	R	64	63.9	…	0.014	15	…
Leschka[73]	2008	Switzerland	2.077	61.9	Siemens	83	R	64	67.7	720	0.021	15	7–9
Maruyama[74]	2008	Japan	2.73	69.1	GE	175	R	64	56.1	…	0.045	15	21.1
Maruyama[74]	2008	Japan	1.62	69.9	GE	175	P	64	54.6	…	0.034	15	4.3
Miller[75]	2008	Multi-countries	2.83	59	Toshiba	200	R	64	60	80	…	19	13.8–15.2
Pugliese[76]	2008	Netherlands	3.25	59	Siemens	165	R	64	58	440	0.026	17	15–20
Pundziute[77]	2008	Netherlands	0.98	60	Toshiba	200	R	64	…	216	…	17	…
Ravipati[78]	2008	United States	1.94	66	Siemens	83	…	64	…	…	…	…	…
Scheffel[79]	2008	Switzerland	1.44	68	Siemens	83	P	64	59	238	0.017	…	1.6
Stolzmann[80]	2008	Switzerland	0.72	65.8	Siemens	83	P	64	60.7	316	0.04	16	2.6
Dewey[81]	2009	Germany	2.63	61	Toshiba	175	P	320	59.9	384	0	16	4.2
Gaudio[82]	2009	Italy	1.68	65	Siemens	165	R	64	…	…	…	…	10.6
Herzog[83]	2009	Switzerland	2.23	62	GE	165	P	64	55.4	…	0.0282	16	2.1
Leschka[84]	2009	United States	4	62	Siemens	75	P	128	58	…	0.01	16	0.9
Meng[85]	2009	China	1.66	63	Siemens	83	…	64	71.8	821	0.02	15	…
Pontone[86]	2009	Italy	7	64.8	GE	175	P	64	54.7	375	0.04	15	5.7
Pontone[86]	2009	Italy	4.33	64.3	GE	175	R	64	57.4	334	0.03	…	20.5
Reimann[87]	2009	Germany	2.75	62	Siemens	83	R	64	62.7	707	0	13	…
Rixe[88]	2009	Germany	1.62	65	Siemens	83	R	64	68	337	0.007	16	13.8–14.3
Sheikh[89]	2009	Kuwait	4.62	60	GE	165	R	64	…	…	…	13	…
Weustink[29]	2009	Switzerland	2.24	61.5	Siemens	165	R	64	69.8	…	…	17	14.2
Weustink[29]	2009	Switzerland	2.21	61.9	Siemens	165	P	64	68.8	…	…	17	10.7
Alkadhi[33]	2010	Switzerland	3.17	62	Siemens	75	P	128	58	…	0.014	16	1.4
Alkadhi[33]	2010	Switzerland	2.57	63	Siemens	75	P	128	56	…	0.011	16	0.9
Andreini[90]	2010	Italy	7.08	65.4	GE	165	R	64	61.2	479	…	15	14.3
Andreini[90]	2010	Italy	7.08	63.3	GE	165	R	64	58	356	…	15	…
Cademartiri[91]	2010	Italy	1.13	53.6	Siemens	165	R	64	58	151	…	15	15–21
Cademartiri[92]	2010	Italy	0.52	48	Siemens	165	R	64	59.4	…	…	17	15–22
Carrascosa[93]	2010	United States	1.94	62.4	Philips	200	P	64	54.9	…	0.021	17	3.4
Chen[94]	2010	China	1.62	60.7	Siemens	83	…	64	86.4	…	0.014	15	…
de Graaf[95]	2010	Netherlands	1.13	61	Toshiba	175	P	320	60	184	0.01	17	3.9–6
Donati[96]	2010	Switzerland	4.22	64	Siemens	83	R	64		…	0.005	16	2.5
Fang[97]	2010	China	1.78	59.6	Siemens	83	R	64	88	…	0.012	15	…
Husmann[98]	2010	Switzerland	1.54	61	GE	175	P	64	56	481	…	16	2.1
Kajander[99]	2010	Finland	1.49	63.5	GE	175	R	64	…	…	…	17	7.6
Nasis[100]	2010	Australia	1.52	63.2	Toshiba	175	P	320	65	…	0.02	17	5.4
Nazeri[101]	2010	Iran	1.68	58	Siemens	165	R	64	62	…	0	15	…
Ovrehus[102]	2010	Denmark	1.28	61	Siemens	83	…	64	61	…	0.025	16	8.4
Sato[103]	2010	Japan.	2.33	67	Toshiba	200	R	64	…	…	…	…	…
Scheffel[104]	2010	Switzerland	2.45	56	Siemens	83	…	64	67.3	126	0.009	16	…
Scheffel[105]	2010	Switzerland	3.78	64	Siemens	83	P	64	61	…	0.014	16	…
Xu[106]	2010	China	2.23	64.6	Siemens	83	R	64	71.4	…	…	15	16.1
Yang[107]	2010	China	3.6	62	Siemens	83	…	64	59	…	…	15	…
Zhang[108]	2010	China	2.65	64	Siemens	165	R	64	76	136.7	0.038	15	16.51
Achenbach[109]	2011	Germany	2.13	59	Siemens	75	P	128	71	…	0	…	0.76
Bamberg[110]	2011	Multi-countries	…	68.1	Siemens	75	P	128	72.2		0.04	17	3.1
Gang[111]	2011	China	1.73	68	Toshiba	175	R	320	73.7	653	0.021	15	12.5
Kerl[112]	2011	United States	1.31	65	Siemens	165	R	64	66	…	0.028	15	…
Moon[113]	2011	Korea	1.85	60.5	Siemens	83	R	64	58.9	…	0.02	15	5.8
Stolzmann[114]	2011	Switzerland	1.38	68	Siemens	83	P	64	58	…	0.016	16	2.2
van Velzen[115]	2011	Netherlands	1.78	57	Toshiba	175	P	320/64	…	…	0.04	17	3.2–7.1
van Velzen[116]	2011	Netherlands	2.03	57	Toshiba	175	P	320	58	139	0.036	17	6–12
Vavere[117]	2011	Multi-countries	3.03	61	Toshiba	175	R	64	61	…	0.096	19	…
Xu[118]	2011	China	0.76	60.4	Toshiba	175	P	320	60.4	…	0.03	15	13
Zhang[119]	2011	China	…	…	Toshiba	200	R	64	60.5	…	…	15	19.88
Zhang[119]	2011	China	…	…	Toshiba	175	P	320	60.9	…	…	15	4.27
Dharampal[120]	2012	Netherlands.	1.18	58	Siemens	165	…	128	66	252	…	17	12
Kadokami[121]	2012	Japan.	2.5	70	Toshiba	200	…	640	70	…	…	15	16.
Kerl[122]	2012	Germany	1.08	67	Siemens	165	R	64	64	…	…	15	…
Maffei[123]	2012	Italy	1.13	61.2	Siemens	75	R	128	64.3	178	…	17	8.9
Maffei[124]	2012	Italy	1.8	59.3	Siemens	75	…	64	58	444	…	17	…
Sohns[125]	2012	Germany.	2.18	57	Siemens	…	R	64	84		…	15	…
Uehara[126]	2012	…	2.12	64.4	Toshiba	175	…	320	65.2	180	…	15	…
van Velzen[127]	2012	Netherlands.	2.03	57	Toshiba	175	P	64	58	…	0.04	17	12.0
Gueret[128]	2013	France	2.44	61	GE/Philips	…	…	64	63	396	…	…	17.2
Pelliccia[129]	2013	Italy	1.95	61	Toshiba	175	P	64	…	…	…	16	…

Note. —ECG = electrocardiographic, R = retrospective, P = prospective, HR = heart rate.

### Methodological Quality

The inter-observer agreement for assessing quality items was good (κ = 0.83). According to the QUADAS tool, 62 studies had a quality score of ≧10 and 29 studies had a quality score of <10. Table S1 demonstrates the QUADAS quality of the included studies.

### Data Synthesis and Statistical Analysis

A total of 126,615 segments, 21,834 vessels, and 9,696 patients were analyzed. The publication bias was significant at the patient, artery and segment levels (P = 0.004, 0.001 and 0.005 respectively). The pooled positive and negative LRs at patient level were 8.91 (95% CI, 7.53, 10.54) and 0.02 (CI, 0.01, 0.03), respectively. At the patient level, significant heterogeneity was detected for positive and negative LRs (Q = 976.63; *P*<0.001; I^2^ = 88.58% [CI, 88.58%, 91.55%]; Q = 476.01; *P*<0.001; I^2^ = 79.62% [CI, 75.88%, 83.37%], respectively).

**Figure 2:**
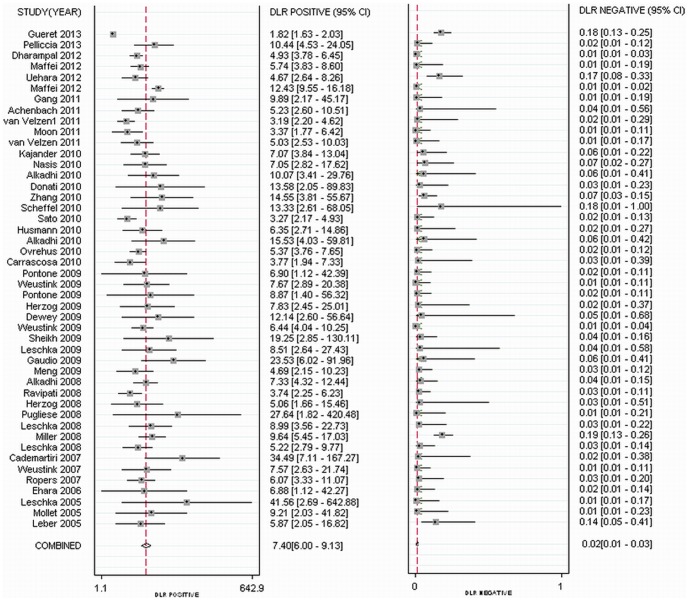
Forest plots showing positive and negative LRs at the patient level. For studies including uninterpretable results, the pooled positive LR was 7.40 (CI, 6.00, 9.13) and negative LR was 0.02 (CI, 0.01, 0.03), respectively.

As methodological quality may significantly influence diagnostic accuracy, we investigated the impact of QUADAS items on the heterogeneity. We found that item 13 was the only key factor for heterogeneity (P = 0.02). In the present study, item 13 was defined as, “How authors handled uninterpretable results.” For studies that did not claim that non-evaluable segments were included, the pooled positive and negative LRs at patient level were 11.16 (CI, 8.90, 14.00) and 0.01 (CI, 0.01, 0.03), respectively. Slight heterogeneity was detected for positive and negative LRs (Q = 67.06; *P*<0.001; I^2^ = 89.78% [CI, 89.78%, 92.36%]; Q = 78.23; *P*<0.001; I^2^ = 38.64% [CI, 17.37%, 59.91%], respectively). For studies including uninterpretable results, the diagnostic performance decreased, with the pooled positive LR 7.40 (CI, 6.00, 9.13) and negative LR 0.02 (CI, 0.01, 0.03). Moderate heterogeneity was found for positive LR (Q = 705.71; *P*<0.001; I^2^ = 91.75% [CI, 91.75%, 94.37%]) and significant heterogeneity for negative LR (Q = 346.89; *P*<0.001; I^2^ = 85.87% [CI, 82.59%, 89.16%]) (Figure 2). The detailed diagnostic accuracy at patient, artery and segment levels is presented in Table 2 as well as in Table S2.

**Table 2:** Overall Diagnostic Performance of CT Angiography.

	All references		References excluding non-diagnostic image		References including non-diagnostic image	
	Positive LR(95% CI)	Negative LR(95% CI)	Positive LR(95% CI)	Negative LR(95% CI)	Positive LR(95% CI)	Negative LR(95% CI)
Patient level	8.91(7.53, 10.54)	0.02 (0.01, 0.03)	11.16(8.90, 14.00)	0.01(0.01, 0.03)	7.40(6.00, 9.13)	0.02(0.01, 0.03)
Artery level	15.22(12.44, 18.64)	0.05(0.04, 0.07)	16.27(12.37, 21.42)	0.05(0.03, 0.08)	14.45(10.83, 19.27)	0.05(0.04, 0.08)
Segment level	31.57(26.92, 37.02)	0.08(0.07, 0.10)	39.76(31.84, 49.63)	0.08(0.06, 0.11)	23.91(19.62, 29.14)	0.08(0.07, 0.11)

Note. —LR =  likelihood ratio.

Using the pre-specified potential sources of heterogeneity as covariates in the random effects models, we found that gender, heart rate, scheme of the coronary arterial tree for stenostic analysis, temporal resolution, calcium score, the proportion of non-assessable segments, and the protocol of ECG-triggered scanning were significant predictors, while age did not impact diagnostic accuracy (Table S3). The results for the subgroups in patient-based analyses are shown in Table 3.

**Table 3:** Pooled Summary Results by Subgroups.

	Patient level (95% CI)		Artery level (95% CI)		Segment level (95% CI)		
	Positive LR	Negative LR	Positive LR	Negative LR	Positive LR	Negative LR	
Temporal resolution							
<100ms	7.23(5.74, 9.09)	0.02(0.01, 0.03)	14.21(9.86, 20.48)	0.05(0.03, 0.07)	25.55(19.49, 33.50)	0.08(0.06, 0.10)	
>100ms	7.83(6.03, 10.16)	0.02(0.01, 0.03)	15.61(10.29, 23.66)	0.05(0.03, 0.10)	23.01(17.60, 30.09)	0.08(0.06, 0.12)	
ECG-triggered protocol							
Retrospective	8.41(5.83, 12.13)	0.02(0.01, 0.04)	17.10(10.71, 27.30)	0.06(0.03, 0.12)	23.74(18.79, 30.00)	0.08(0.05, 0.12)	
Prospective	5.95(4.52, 7.84)	0.03(0.02, 0.06)	12.41(8.22, 18.72)	0.05(0.03, 0.08)	25.64(17.895, 36.77)	0.107(0.07, 0.13)	
Gender							
Male/female<3	7.01(5.59, 8.80)	0.02(0.01, 0.03)	16.52(12.43, 21.95)	0.05(0.03, 0.08)	25.148(19.58, 32.30)	0.08(0.06, 0.12)	
Male/female>3	11.02(6.56, 18.52)	0.02(0.01, 0.04)	9.43(4.49, 19.82)	0.05(0.03, 0.09)	19.87(14.20, 27.81)	0.08(0.05, 0.12)	
Scheme of coronary tree							
≦16-segments	7.82(6.17, 9.93)	0.02(0.01, 0.04)	16.75(11.07, 25.34)	0.06(0.04, 0.08)	24.89(18.81, 32.93)	0.09(0.07, 0.12)	
>16-segments	7.33(5.53, 9.72)	0.01(0.00, 0.03)	12.28(8.65, 17.44)	0.05(0.02, 0.12)	22.50(16.57, 30.54)	0.06(0.05, 0.08)	
Heart rate							
<65bpm	7.55(5.52, 10.34)	0.02(0.01, 0.04)	13.09(9.32, 18.40)	0.06(0.04, 0.11)	21.79(17.46, 27.19)	0.10(0.07, 0.14)	
>65bpm	6.89(5.28, 8.98)	0.02(0.01, 0.04)	18.07(9.48, 34.47)	0.03(0.02, 0.07)	26.11(18.36, 37.14)	0.06(0.05, 0.09)	
Calcium score							
<400	7.23(5.86, 8.92)	0.02(0.01, 0.03)	13.97(8.22, 23.72)	0.06(0.03, 0.15)	22.36(15.31, 32.65)	0.08(0.04, 0.15)	
>400	11.51(9.13, 14.53)	0.01(0.01, 0.02)	16.37(7.00, 38.30)	0.04(0.02, 0.09)	19.43(12.28, 30.72)	0.06(0.03, 0.12)	
Prevalence of CAD							
<0.5	7.50 (5.32, 10.58)	0.05(0.02, 0.08)	18.27(13.11, 25.48)	0.06(0.03, 0.11)	28.35(21.32, 37.71)	0.09(0.06, 0.15)	
>0.5	7.18(5.62, 9.17)	0.01(0.01, 0.02)	11.48(7.473, 17.64)	0.05(0.03, 0.07)	20.06(15.36, 26.21)	0.08(0.06, 0.11)	
Prevalence of non-assessable segments							
<0.02	9.12(6.41, 12.96)	0.03(0.01, 0.05)	18.73(13.76, 25.50)	0.05(0.03, 0.08)	33.303(25.574, 43.369)	0.08(0.05, 0.11)	
>0.02	6.36(4.32, 9.36)	0.01(0.01, 0.04)	12.21(7.19, 20.74)	0.04(0.02, 0.07)	17.62(14.14, 21.94)	0.09(0.06, 0.12)	

Note. —TP = true-positive, FP  =  false-positive, TN = true-negative, FN =  false-negative, LR = likelihood ratio, ECG = electrocardiographic, bpm = beat per minute, BMI = body mass index, CAD = coronary artery disease.

**Figure 3:**
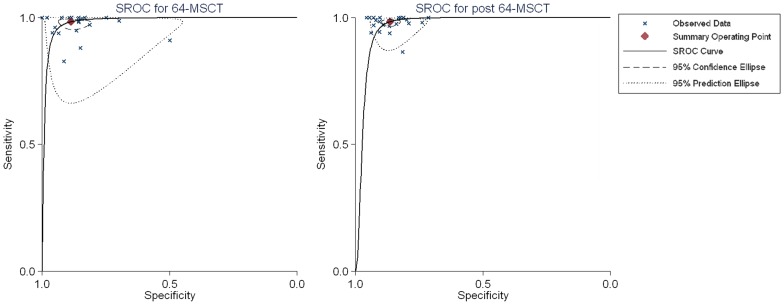
sROC for 64-MDCT and post 64-MDCT. sROC demonstrated the diagnostic performance of 64-MDCT and post 64-MDCT at the patient level (sROC =  summary receiver operating characteristic).

We added the generation of CT scanners as a covariate to the bivariate model to compare the performance of 64-MDCT and post 64-MDCT. The result showed that, with new techniques used in the newer generations of CT scanners, the overall accuracy of post 64-MDCT at the patient level decreased (P<0.001, the area under the summary ROC curve were 0.98 [CI, 0.97 to 0.99] for 64-MDCT and 0.96 [CI, 0.94 to 0.98] for post 64-MDCT, respectively) (Figure 3). Further analysis indicated that the decreased index was positive LRs (8.71 [5.76, 13.12] versus 7.24 [5.92, 8.85]), while the negative LRs were similar (0.02 [0.01, 0.04] versus 0.02 [0.01, 0.03]). Importantly, the heterogeneity of 64-MDCT was larger than that of post 64-MDCT (I^2^ = 96.26% versus 30.2%; 91.89% versus 56.79% for positive and negative LRs, respectively).

**Figure 4:**
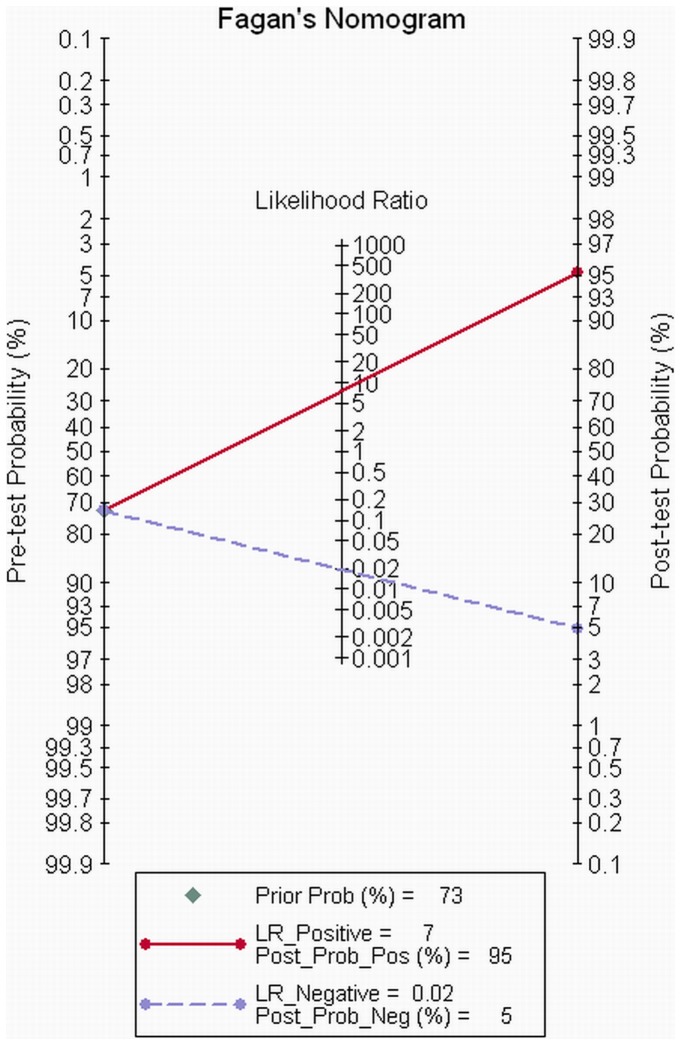
Fagan's nomogram for CTA. For patients with a pre-test probability of CAD<73%, the post-test probability of negative results was larger than 95%. However, when the pre-test probability was larger than 73%, the diagnostic role was reversed, with a positive post-test probability of larger than 95% and a negative post-test probability of less than 95%.

We evaluated the post-test probability for studies that explicitly stated that the non-assessable segments were included during analysis, as it is common practice to classify a segment as diseased if there is any doubt. The relationship between pre-test probability and post-test probability was depicted by visual Fagan's nomogram [23]. As we can see from Figure 4, for patients with a pre-test probability of CAD<73%, the post-test probability of negative results was larger than 95%, while the post-test probability of positive results was less than 95%, which indicated that the CT angiography was only an effective tool to exclude patients with CAD. On the other hand, when the pre-test probability was larger than 73%, the diagnostic role was reversed, with a positive post-test probability of larger than 95% and a negative post-test probability of less than 95%, which implied that when there was a pre-test probability of CAD >73%, the role of CTA changed from a test of exclusion to a confirmatory tool.

**Figure 5:**
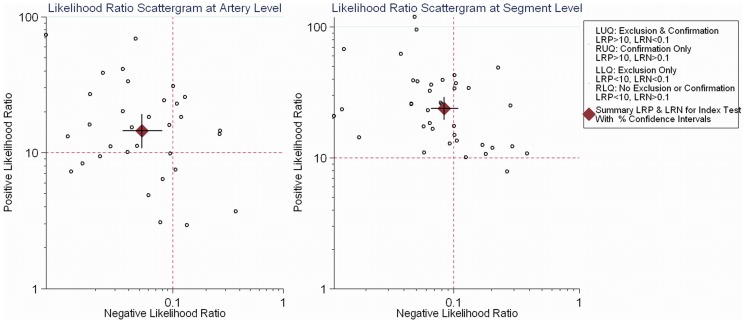
Illustration chart indicating the diagnostic role for accuracy of CTA at the artery and segment levels. At the artery and segment levels, the likelihood ratio profile of CT was both an exclusion and s confirmation test to diagnose stenosis >50% (positive likelihood ratio >10; negative likelihood ratio <0.1), while CTA was generally a test of exclusion to rule out significant stenosis at the patient level (LRP =  positive likelihood ratio, LRN  =  negative likelihood ratio, LLQ  =  left lower quadrant, LUQ  =  left upper quadrant, RLQ  =  right lower quadrant, RUQ  =  right upper quadrant).

The likelihood ratio scattergram showed that at the artery and segment levels, the likelihood ratio profile of CTA was both a test of exclusion and a confirmatory test tool to diagnose stenosis >50% (positive likelihood ratio >10; negative likelihood ratio <0.1), while CTA was generally a test of exclusion to rule out significant stenosis at the patient level (positive likelihood ratio <10; negative likelihood ratio <0.1) (Figure 5) [24].

The sensitivity analysis was conducted at the patient level to investigate the influence of each individual study on the overall meta-analysis summary estimate. No study influenced the pooled sensitivity and specificity larger than 0.02 (Figure S1).

## Discussion

The present study analyzed studies of 64-MDCT and post 64-MDCT to shed new light on the following critical questions: (1) What is the role of CTA in patients with different pre-test probabilities? (2) Can post 64-MDCT lead to a better diagnostic accuracy than 64-MDCT?

The hypothesis, which states that CTA can exclude individuals with suspected CAD for patients with low to intermediate pre-test likelihood, has been argued widely in previous studies [3], [20]. The present study showed that not all patients with an intermediate pre-test likelihood of CAD could be excluded by CTA. After assessing the precise analysis based on Bayes' theorem, the results show that the pre-test probability of 73% is the cut off value for the diagnostic role of CTA. When pre-test probability is <73%, CTA is an effective tool to exclude CAD. Of note, the positive LR is comparatively low and CTA may be still applied to determine the presence of obstructive CAD in patients with a pre-test probability of CAD>73%. The confirmatory application of CTA also plays an important role for clinical diagnosis, especially for patients with stable angina when revascularization is not preferred at the present time [25]. CTA, therefore, may provide more evidence to confirm the presence of CAD and avoid the major complications associated with ICA [2], [25]. As there are several studies providing detailed algorithms to quantify pre-test risk [9]–[12], a precise evaluation of the diagnostic role of CTA according to pre-test probability will improve the cost-effectiveness of CTA. Moreover, the present analysis demonstrates that CTA is both a test of exclusion and confirmatory test on both artery and segment levels, which provides detailed information for prognostic evaluation [26], [27] or for the selection of a revascularization strategy [28].

The results indicate that the exclusive performance of post 64-MDCT does not increase. Rather, the confirmatory accuracy of post 64-MDCT decreases. The main reason may be that the improved techniques mainly focuses on the motion artifacts. For example, DSCT, with two detectors arranged at angles of 90°, effectively doubles the temporal resolution [29]. Additionally, 320-MDCT, with 16-cm wide volume coverage, enabled whole coronary arteries covered with one heartbeat, which eliminated the motion artifact effectively [30]. The spatial resolution, however, is not improved and the beam hardening artifacts still present a primary shortcoming of CTA. As is apparent from the results, the heterogeneity of post 64-MDCT was significantly larger than that of 64-MDCT. The decreased positive LR of post 64-MDCT may be caused by a different prevalence of calcified plaques, which are not, however, provided by most studies. Further analysis with information regarding the individual may illustrate the question more clearly. Another potential reason for the decreased pooled positive LR may be that with an improved technique of post 64-MDCT, the inclusion criteria of patients was broader than that of 64-MDCT. With dual-source, 64-slice CT, it could be theoretically possible to scan without lowering the heart rate [31]. Nevertheless, in reality, the heart rate still needs to remain low for good image quality since the temporal resolution of this scanner is still too low. In addition, a low heart rate is also suggested for 128-MDCT or 320-MDCT due to the technical specifications of the scanners. Nevertheless, with wider detection, prospective ECG gating is more feasible. Combined with iterative construction, the radiation dose of post 64-MDCT was significantly lower than that of 64-MDCT [32], [33].

Schueler et al. reported that item referring to how authors handled “Uninterpretable Results” (QUADAS item 13) had a significant influence on diagnostic accuracy values and that exclusion of uninterpretable results may overestimate the diagnostic abilities of the method being investigated[34], which has also been proven by our analysis. Salavati et al., on the other hand, did not detect a difference between studies including and excluding uninterpretable segments[35]. The potential reason might be the lower proportion of uninterpretable segments achieved by DSCT[35]. The present study, with 64- and post 64-MDCT included, evaluates the diagnostic performance mainly through studies that clearly claim that coronary arterial segments with non-diagnostic image quality were treated as positive for disease, as it is a common practice to classify a segment as diseased if it is non-diagnostic, which guarantees the validity of the conclusion reached.

Our meta-analysis has limitations. First, we did not analyze the diagnostic accuracy of CTA for stable angina and ACS respectively, as most studies include all symptomatic patients without further classification of clinical features. In addition, though the clinical diagnoses were different, the morphologic images of angiography were both indicative of stenosis of the coronary lumen. Second, although the present study is aimed at illustrating the diagnostic role of CTA by pre-test probability, information on symptoms, however, are not provided by most references. However, the main conclusion was not impacted as we mainly used Bayes' theorem and the positive and negative LRs, instead of the sensitivity and specificity, to evaluate the diagnostic role of CT angiography. Such LRs have advantages for the following reasons: they are less likely to change with the prevalence of CAD and they can be calculated for several levels of the symptom/sign. Third, the conclusion was conducted by using ICA as standard references. Recent studies indicate that fractional flow reserve (FFR)-guided treatment may lead to a better prognosis than that of an ICA-guided strategy [36], [37]. Several studies, however, demonstrate that the accuracy to diagnose ischemic stenosis, with FFR as a reference, was poor [38]-[40]. Further meta-analysis is therefore required to explore the different capacities of CTA for detecting morphological and functional stenosis.

In conclusion, the diagnostic performance of post 64-MDCT does not increase as compared with 64-MDCT. CTA, overall, is a test of exclusion for patients with a pre-test probability of CAD<73%, while for patients with a pre-test probability of CAD>73%, CTA is a test used to confirm the presence of CAD.

## Supporting Information

Figure S1 **The Serrbar Illustrating a Sensitivity Analysis in which the Meta-Analysis was Re-estimated by Omitting Each Study in Turn.** The sensitivity analysis indicates that no study influenced the pooled sensitivity and specificity larger than 0.02.
(TIF)

Table S1 **Quality Assessment of Studies Enrolled to Diagnostic Accuracy.**
(DOCX)

Table S2 **Detailed Diagnostic Information for Each Study.**
(DOCX)

Table S3 **Results of the Multivariate Meta-Regression Analysis for Identifying Covariates to Explain Heterogeneity at Patient Level.**
(DOCX)

Checklist S1 **PRISMA Checklist.**
(DOC)

## References

[1] Naghavi M, Libby P, Falk E, Casscells SW, Litovsky S, et al (2003) From vulnerable plaque to vulnerable patient: a call for new definitions and risk assessment strategies: Part I. Circulation 108: 1664–1672. 14530185 10.1161/01.CIR.0000087480.94275.97

[2] Mowatt G, Cummins E, Waugh N, Walker S, Cook J, et al. (2008) Systematic review of the clinical effectiveness and cost-effectiveness of 64-slice or higher computed tomography angiography as an alternative to invasive coronary angiography in the investigation of coronary artery disease. Health Technol Assess 12 : iii-iv, ix-143. 10.3310/hta12170 18462576

[3] Schuetz GM, Zacharopoulou NM, Schlattmann P, Dewey M (2010) Meta-analysis: noninvasive coronary angiography using computed tomography versus magnetic resonance imaging. Annals of internal medicine 152: 167–177. 20124233 10.7326/0003-4819-152-3-201002020-00008

[4] Hamon M, Morello R, Riddell JW (2007) Coronary arteries: diagnostic performance of 16- versus 64-section spiral CT compared with invasive coronary angiography–meta-analysis. Radiology 245: 720–731. 17951354 10.1148/radiol.2453061899

[5] Vanhoenacker PK, Heijenbrok-Kal MH, Van Heste R, Decramer I, Van Hoe LR, et al (2007) Diagnostic performance of multidetector CT angiography for assessment of coronary artery disease: meta-analysis. Radiology 244: 419–428. 17641365 10.1148/radiol.2442061218

[6] Schlattmann P, Schuetz GM, Dewey M (2011) Influence of coronary artery disease prevalence on predictive values of coronary CT angiography: a meta-regression analysis. Eur Radiol 21: 1904–1913. 21597986 10.1007/s00330-011-2142-2

[7] von Ballmoos MW, Haring B, Juillerat P, Alkadhi H (2011) Meta-analysis: diagnostic performance of low-radiation-dose coronary computed tomography angiography. Annals of internal medicine 154: 413–420. 21403076 10.7326/0003-4819-154-6-201103150-00007

[8] Weustink AC, de Feyter PJ (2011) The role of multi-slice computed tomography in stable angina management: a current perspective. Neth Heart J 19: 336–343. 21792743 10.1007/s12471-011-0096-2 PMC3144326

[9] Budaj A, Dean V, Deckers J, Dickstein K (2006) Guidelines on the management of stable angina pectoris. European Heart Journal: 1–63. 10.1093/eurheartj/ehl257 17028111

[10] Cheng VY, Berman DS, Rozanski A, Dunning AM, Achenbach S, et al. (2011) Performance of the traditional age, sex, and angina typicality-based approach for estimating pretest probability of angiographically significant coronary artery disease in patients undergoing coronary computed tomographic angiography: results from the multinational coronary CT angiography evaluation for clinical outcomes: an international multicenter registry (CONFIRM). Circulation 124: 2423–2432, 2421–2428. 10.1161/CIRCULATIONAHA.111.039255 PMC3240578 22025600

[11] Pryor DB, Shaw L, McCants CB, Lee KL, Mark DB, et al (1993) Value of the history and physical in identifying patients at increased risk for coronary artery disease. Annals of internal medicine 118: 81–81. 8416322 10.7326/0003-4819-118-2-199301150-00001

[12] Diamond GA, Forrester JS (1979) Analysis of probability as an aid in the clinical diagnosis of coronary-artery disease. N Engl J Med 300: 1350–1358. 440357 10.1056/NEJM197906143002402

[13] Moher D, Liberati A, Tetzlaff J, Altman DG (2009) Preferred reporting items for systematic reviews and meta-analyses: the PRISMA statement. Ann Intern Med 151: : 264–269, W264. PMC3090117 21603045

[14] Whiting P, Rutjes A, Reitsma J, Bossuyt P, Kleijnen J (2003) The development of QUADAS: a tool for the quality assessment of studies of diagnostic accuracy included in systematic reviews. BMC medical research methodology 3: 1–13. 14606960 10.1186/1471-2288-3-25 PMC305345

[15] Deeks JJ, Macaskill P, Irwig L (2005) The performance of tests of publication bias and other sample size effects in systematic reviews of diagnostic test accuracy was assessed. Journal of clinical epidemiology 58: 882–893. 16085191 10.1016/j.jclinepi.2005.01.016

[16] Higgins J, Thompson SG, Deeks JJ, Altman DG (2003) Measuring inconsistency in meta-analyses. Bmj 327: 557. 12958120 10.1136/bmj.327.7414.557 PMC192859

[17] Swets JA (1988) Measuring the accuracy of diagnostic systems. Science 240: 1285–1293. 3287615 10.1126/science.3287615

[18] Chu H, Cole SR, Reitsma J, Zwinderman A (2006) Bivariate meta-analysis of sensitivity and specificity with sparse data: a generalized linear mixed model approach. Authors' reply. Journal of clinical epidemiology 59: 1331–1333. 17098577 10.1016/j.jclinepi.2006.06.011

[19] Reitsma JB, Glas AS, Rutjes AWS, Scholten RJPM, Bossuyt PM, et al (2005) Bivariate analysis of sensitivity and specificity produces informative summary measures in diagnostic reviews. Journal of clinical epidemiology 58: 982–990. 16168343 10.1016/j.jclinepi.2005.02.022

[20] von Ballmoos MW, Haring B, Juillerat P, Alkadhi H (2011) Meta-analysis: diagnostic performance of low-radiation-dose coronary computed tomography angiography. Ann Intern Med 154: 413–420. 21403076 10.7326/0003-4819-154-6-201103150-00007

[21] Skupski DW, Rosenberg CR, Eglinton GS (2002) Intrapartum Fetal Stimulation Tests: A Meta-Analysis. Obstetrics & Gynecology 99: 129–134. 11777523 10.1016/s0029-7844(01)01645-3

[22] Jaeschke R, Guyatt GH, Sackett DL, Guyatt G, Bass E, et al (1994) Users' guides to the medical literature. JAMA: the journal of the American Medical Association 271: 703–707. 8309035 10.1001/jama.271.9.703

[23] Fagan TJ (1975) Letter: Nomogram for Bayes theorem. The New England journal of medicine 293: 257. 1143310 10.1056/NEJM197507312930513

[24] Stengel D, Bauwens K, Sehouli J, Ekkernkamp A, Porzsolt F (2003) A likelihood ratio approach to meta-analysis of diagnostic studies. J Med Screen 10: 47–51. 12790315 10.1258/096914103321610806

[25] Moscariello A, Vliegenthart R, Schoepf UJ, Nance JW Jr, Zwerner PL, et al (2012) Coronary CT angiography versus conventional cardiac angiography for therapeutic decision making in patients with high likelihood of coronary artery disease. Radiology 265: 385–392. 22875799 10.1148/radiol.12112426

[26] Andreini D, Pontone G, Mushtaq S, Bartorelli AL, Bertella E, et al (2012) A long-term prognostic value of coronary CT angiography in suspected coronary artery disease. JACC: Cardiovascular Imaging 5: 690–701. 22789937 10.1016/j.jcmg.2012.03.009

[27] Nance JW, Jr., Schlett CL, Schoepf UJ, Oberoi S, Leisy HB, et al. (2012) Incremental prognostic value of different components of coronary atherosclerotic plaque at cardiac CT angiography beyond coronary calcification in patients with acute chest pain. Radiology. 10.1148/radiol.12112350 22820732

[28] Moscariello A, Vliegenthart R, Schoepf UJ, Nance JW, Jr., Zwerner PL, et al. (2012) Coronary CT angiography versus conventional cardiac angiography for therapeutic decision making in patients with high likelihood of coronary artery disease. Radiology. 10.1148/radiol.12112426 22875799

[29] Weustink AC, Mollet NR, Neefjes LA, van Straten M, Neoh E, et al (2009) Preserved diagnostic performance of dual-source CT coronary angiography with reduced radiation exposure and cancer risk. Radiology 252: 53–60. 19451542 10.1148/radiol.2521082023

[30] Chen MY, Shanbhag SM, Arai AE (2013) Submillisievert median radiation dose for coronary angiography with a second-generation 320-detector row CT scanner in 107 consecutive patients. Radiology 267: 76–85. 23340461 10.1148/radiol.13122621 PMC3606544

[31] Hsiao EM, Rybicki FJ, Steigner M (2010) CT coronary angiography: 256-slice and 320-detector row scanners. Curr Cardiol Rep 12: 68–75. 20425186 10.1007/s11886-009-0075-z PMC2893879

[32] Yoneyama K, Vavere AL, Cerci R, Ahmed R, Arai AE, et al (2012) Influence of image acquisition settings on radiation dose and image quality in coronary angiography by 320-detector volume computed tomography: the CORE320 pilot experience. Heart Int 7: e11. 23185678 10.4081/hi.2012.e11 PMC3504303

[33] Alkadhi H, Stolzmann P, Desbiolles L, Baumueller S, Goetti R, et al (2010) Low-dose, 128-slice, dual-source CT coronary angiography: accuracy and radiation dose of the high-pitch and the step-and-shoot mode. Heart 96: 933–938. 20538669 10.1136/hrt.2009.189100

[34] Schueler S, Walther S, Schuetz GM, Schlattmann P, Dewey M (2013) Methodological quality of diagnostic accuracy studies on non-invasive coronary CT angiography: influence of QUADAS (Quality Assessment of Diagnostic Accuracy Studies included in systematic reviews) items on sensitivity and specificity. Eur Radiol 23: 1603–1622. 23322410 10.1007/s00330-012-2763-0

[35] Salavati A, Radmanesh F, Heidari K, Dwamena BA, Kelly AM, et al. (2011) Dual-source computed tomography angiography for diagnosis and assessment of coronary artery disease: Systematic review and meta-analysis. J Cardiovasc Comput Tomogr. 10.1016/j.jcct.2011.10.018 22226727

[36] Tonino PA, De Bruyne B, Pijls NH, Siebert U, Ikeno F, et al (2009) Fractional flow reserve versus angiography for guiding percutaneous coronary intervention. N Engl J Med 360: 213–224. 19144937 10.1056/NEJMoa0807611

[37] Pijls NH, Fearon WF, Tonino PA, Siebert U, Ikeno F, et al (2010) Fractional flow reserve versus angiography for guiding percutaneous coronary intervention in patients with multivessel coronary artery disease: 2-year follow-up of the FAME (Fractional Flow Reserve Versus Angiography for Multivessel Evaluation) study. J Am Coll Cardiol 56: 177–184. 20537493 10.1016/j.jacc.2010.04.012

[38] Meijboom WB, Van Mieghem CA, van Pelt N, Weustink A, Pugliese F, et al (2008) Comprehensive assessment of coronary artery stenoses: computed tomography coronary angiography versus conventional coronary angiography and correlation with fractional flow reserve in patients with stable angina. J Am Coll Cardiol 52: 636–643. 18702967 10.1016/j.jacc.2008.05.024

[39] Ko BS, Cameron JD, Meredith IT, Leung M, Antonis PR, et al (2012) Computed tomography stress myocardial perfusion imaging in patients considered for revascularization: a comparison with fractional flow reserve. Eur Heart J 33: 67–77. 21810860 10.1093/eurheartj/ehr268

[40] Min JK, Leipsic J, Pencina MJ, Berman DS, Koo BK, et al (2012) Diagnostic accuracy of fractional flow reserve from anatomic CT angiography. JAMA 308: 1237–1245. 22922562 10.1001/2012.jama.11274 PMC4281479

[41] Leber AW, Knez A, von Ziegler F, Becker A, Nikolaou K, et al (2005) Quantification of Obstructive and Nonobstructive Coronary Lesions by 64-Slice Computed Tomography: A Comparative Study With Quantitative Coronary Angiography and Intravascular Ultrasound. Journal of the American College of Cardiology 46: 147–154. 15992649 10.1016/j.jacc.2005.03.071

[42] Leschka S, Alkadhi H, Plass A, Desbiolles L, Grunenfelder J, et al (2005) Accuracy of MSCT coronary angiography with 64-slice technology: first experience. Eur Heart J 26: 1482–1487. 15840624 10.1093/eurheartj/ehi261

[43] Mollet NR, Cademartiri F, van Mieghem CAG, Runza G, McFadden EP, et al (2005) High-resolution spiral computed tomography coronary angiography in patients referred for diagnostic conventional coronary angiography. Circulation 112: 2318–2323. 16203914 10.1161/CIRCULATIONAHA.105.533471

[44] Pugliese F, Mollet NRA, Runza G, Mieghem C, Meijboom WB, et al (2005) Diagnostic accuracy of non-invasive 64-slice CT coronary angiography in patients with stable angina pectoris. European radiology 16: 575–582. 16292649 10.1007/s00330-005-0041-0

[45] Raff GL, Gallagher MJ, O'Neill WW, Goldstein JA (2005) Diagnostic accuracy of noninvasive coronary angiography using 64-slice spiral computed tomography. Journal of the American College of Cardiology 46: 552–557. 16053973 10.1016/j.jacc.2005.05.056

[46] Ehara M, Surmely JF, Kawai M, Katoh O, Matsubara T, et al (2006) Diagnostic accuracy of 64-slice computed tomography for detecting angiographically significant coronary artery stenosis in an unselected consecutive patient population: comparison with conventional invasive angiography. Circulation journal: official journal of the Japanese Circulation Society 70: 564–571. 16636491 10.1253/circj.70.564

[47] Ghostine S, Caussin C, Daoud B, Habis M, Perrier E, et al (2006) Non-invasive detection of coronary artery disease in patients with left bundle branch block using 64-slice computed tomography. J Am Coll Cardiol 48: 1929–1934. 17112979 10.1016/j.jacc.2006.04.103

[48] Meijboom WB, Mollet NR, Van Mieghem CA, Kluin J, Weustink AC, et al (2006) Pre-operative computed tomography coronary angiography to detect significant coronary artery disease in patients referred for cardiac valve surgery. J Am Coll Cardiol 48: 1658–1665. 17045904 10.1016/j.jacc.2006.06.054

[49] Nikolaou K, Knez A, Rist C, Wintersperger BJ, Leber A, et al (2006) Accuracy of 64-MDCT in the diagnosis of ischemic heart disease. American Journal of Roentgenology 187: 111–117. 16794164 10.2214/AJR.05.1697

[50] Plass A, Grunenfelder J, Leschka S, Alkadhi H, Eberli F, et al (2006) Coronary artery imaging with 64-slice computed tomography from cardiac surgical perspective. European Journal of Cardio-Thoracic Surgery 30: 109–116. 16725341 10.1016/j.ejcts.2006.03.048

[51] Ropers D, Rixe J, Anders K, Küttner A, Baum U, et al (2006) Usefulness of multidetector row spiral computed tomography with 64-×0.6-mm collimation and 330-ms rotation for the noninvasive detection of significant coronary artery stenoses. The American journal of cardiology 97: 343–348. 16442393 10.1016/j.amjcard.2005.08.050

[52] Scheffel H, Alkadhi H, Plass A, Vachenauer R, Desbiolles L, et al (2006) Accuracy of dual-source CT coronary angiography: First experience in a high pre-test probability population without heart rate control. Eur Radiol 16: 2739–2747. 17031451 10.1007/s00330-006-0474-0 PMC1705545

[53] Schuijf JD, Pundziute G, Jukema JW, Lamb HJ, van der Hoeven BL, et al (2006) Diagnostic accuracy of 64-slice multislice computed tomography in the noninvasive evaluation of significant coronary artery disease. Am J Cardiol 98: 145–148. 16828582 10.1016/j.amjcard.2006.01.092

[54] Cademartiri F, Maffei E, Palumbo A, Malagò R, Alberghina F, et al (2007) Diagnostic accuracy of 64-slice computed tomography coronary angiography in patients with low-to-intermediate risk. La Radiologia Medica 112: 969–981. 17952682 10.1007/s11547-007-0198-5

[55] Herzog C, Zwerner PL, Doll JR, Nielsen CD, Nguyen SA, et al (2007) Significant Coronary Artery Stenosis: Comparison on Per-Patient and Per-Vessel or Per-Segment Basis at 64-Section CT Angiography. Radiology 244: 112–120. 17581898 10.1148/radiol.2441060332

[56] Herzog C, Nguyen SA, Savino G, Zwerner PL, Doll J, et al (2007) Does Two-Segment Image Reconstruction at 64-Section CT Coronary Angiography Improve Image Quality and Diagnostic Accuracy? Radiology 244: 121–129. 17495177 10.1148/radiol.2441060004

[57] Heuschmid M, Burgstahler C, Reimann A, Brodoefel H, Mysal I, et al (2007) Usefulness of noninvasive cardiac imaging using dual-source computed tomography in an unselected population with high prevalence of coronary artery disease. Am J Cardiol 100: 587–592. 17697811 10.1016/j.amjcard.2007.03.066

[58] Johnson TR, Nikolaou K, Busch S, Leber AW, Becker A, et al (2007) Diagnostic accuracy of dual-source computed tomography in the diagnosis of coronary artery disease. Invest Radiol 42: 684–691. 17984765 10.1097/RLI.0b013e31806907d0

[59] Leber AW, Johnson T, Becker A, von Ziegler F, Tittus J, et al (2007) Diagnostic accuracy of dual-source multi-slice CT-coronary angiography in patients with an intermediate pretest likelihood for coronary artery disease. Eur Heart J 28: 2354–2360. 17644815 10.1093/eurheartj/ehm294

[60] Meijboom WB, van Mieghem CA, Mollet NR, Pugliese F, Weustink AC, et al (2007) 64-slice computed tomography coronary angiography in patients with high, intermediate, or low pretest probability of significant coronary artery disease. J Am Coll Cardiol 50: 1469–1475. 17919567 10.1016/j.jacc.2007.07.007

[61] Meijboom WB, Mollet NR, Van Mieghem CA, Weustink AC, Pugliese F, et al (2007) 64-Slice CT coronary angiography in patients with non-ST elevation acute coronary syndrome. Heart 93: 1386–1392. 17344332 10.1136/hrt.2006.112771 PMC2016889

[62] Oncel D, Oncel G, Tastan A, Tamci B (2007) Detection of significant coronary artery stenosis with 64-section MDCT angiography. European journal of radiology 62: 394–405. 17306490 10.1016/j.ejrad.2007.01.009

[63] Ropers U, Ropers D, Pflederer T, Anders K, Kuettner A, et al (2007) Influence of heart rate on the diagnostic accuracy of dual-source computed tomography coronary angiography. J Am Coll Cardiol 50: 2393–2398. 18154964 10.1016/j.jacc.2007.09.017

[64] Scheffel H, Leschka S, Plass A, Vachenauer R, Gaemperli O, et al (2007) Accuracy of 64-slice computed tomography for the preoperative detection of coronary artery disease in patients with chronic aortic regurgitation. Am J Cardiol 100: 701–706. 17697832 10.1016/j.amjcard.2007.03.087

[65] Schlosser T, Mohrs OK, Magedanz A, Nowak B, Voigtlander T, et al (2007) Noninvasive coronary angiography using 64-detector-row computed tomography in patients with a low to moderate pretest probability of significant coronary artery disease. Acta Radiol 48: 300–307. 17453500 10.1080/02841850701203587

[66] Weustink AC, Meijboom WB, Mollet NR, Otsuka M, Pugliese F, et al (2007) Reliable high-speed coronary computed tomography in symptomatic patients. Journal of the American College of Cardiology 50: 786–794. 17707184 10.1016/j.jacc.2007.04.068

[67] Achenbach S, Ropers U, Kuettner A, Anders K, Pflederer T, et al (2008) Randomized comparison of 64-slice single- and dual-source computed tomography coronary angiography for the detection of coronary artery disease. JACC Cardiovasc Imaging 1: 177–186. 19356426 10.1016/j.jcmg.2007.11.006

[68] Alkadhi H, Scheffel H, Desbiolles L, Gaemperli O, Stolzmann P, et al (2008) Dual-source computed tomography coronary angiography: influence of obesity, calcium load, and heart rate on diagnostic accuracy. European Heart Journal 29: 766–776. 18292596 10.1093/eurheartj/ehn044

[69] Brodoefel H, Burgstahler C, Tsiflikas I, Reimann A, Schroeder S, et al (2008) Dual-source CT: effect of heart rate, heart rate variability, and calcification on image quality and diagnostic accuracy. Radiology 247: 346–355. 18372455 10.1148/radiol.2472070906

[70] Han SC, Fang CC, Chen Y, Chen CL, Wang SP (2008) Coronary computed tomography angiography–a promising imaging modality in diagnosing coronary artery disease. J Chin Med Assoc 71: 241–246. 18490228 10.1016/S1726-4901(08)70114-X

[71] Herzog BA, Husmann L, Burkhard N, Gaemperli O, Valenta I, et al (2008) Accuracy of low-dose computed tomography coronary angiography using prospective electrocardiogram-triggering: first clinical experience. European Heart Journal 29: 3037–3042. 18996954 10.1093/eurheartj/ehn485

[72] Leschka S, Scheffel H, Husmann L, Gämperli O, Marincek B, et al (2008) Effect of decrease in heart rate variability on the diagnostic accuracy of 64-MDCT coronary angiography. American Journal of Roentgenology 190: 1583–1590. 18492910 10.2214/AJR.07.2000

[73] Leschka S, Scheffel H, Desbiolles L, Plass A, Gaemperli O, et al (2008) Combining dual-source computed tomography coronary angiography and calcium scoring: added value for the assessment of coronary artery disease. Heart 94: 1154–1161. 18032458 10.1136/hrt.2007.124800

[74] Maruyama T, Takada M, Hasuike T, Yoshikawa A, Namimatsu E, et al (2008) Radiation dose reduction and coronary assessability of prospective electrocardiogram-gated computed tomography coronary angiography: comparison with retrospective electrocardiogram-gated helical scan. Journal of the American College of Cardiology 52: 1450–1455. 19017511 10.1016/j.jacc.2008.07.048

[75] Miller JM, Rochitte CE, Dewey M, Arbab-Zadeh A, Niinuma H, et al (2008) Diagnostic performance of coronary angiography by 64-row CT. N Engl J Med 359: 2324–2336. 19038879 10.1056/NEJMoa0806576

[76] Pugliese F, Mollet NR, Hunink M, Cademartiri F, Nieman K, et al (2008) Diagnostic Performance of Coronary CT Angiography by Using Different Generations of Multisection Scanners: Single-Center Experience. Radiology 246: 384–393. 18180337 10.1148/radiol.2462070113

[77] Pundziute G, Schuijf JD, Jukema JW, van Werkhoven JM, Boersma E, et al (2008) Gender influence on the diagnostic accuracy of 64-slice multislice computed tomography coronary angiography for detection of obstructive coronary artery disease. Heart 94: 48–52. 17540687 10.1136/hrt.2007.116715

[78] Ravipati G, Aronow WS, Lai H, Shao J, DeLuca AJ, et al (2008) Comparison of sensitivity, specificity, positive predictive value, and negative predictive value of stress testing versus 64-multislice coronary computed tomography angiography in predicting obstructive coronary artery disease diagnosed by coronary angiography. Am J Cardiol 101: 774–775. 18328838 10.1016/j.amjcard.2007.10.044

[79] Scheffel H, Alkadhi H, Leschka S, Plass A, Desbiolles L, et al (2008) Low-dose CT coronary angiography in the step-and-shoot mode: diagnostic performance. Heart 94: 1132–1137. 18519548 10.1136/hrt.2008.149971

[80] Stolzmann P, Scheffel H, Leschka S, Plass A, Baumuller S, et al (2008) Influence of calcifications on diagnostic accuracy of coronary CT angiography using prospective ECG triggering. AJR Am J Roentgenol 191: 1684–1689. 19020236 10.2214/AJR.07.4040

[81] Dewey M, Zimmermann E, Deissenrieder F, Laule M, Dubel HP, et al (2009) Noninvasive coronary angiography by 320-row computed tomography with lower radiation exposure and maintained diagnostic accuracy: comparison of results with cardiac catheterization in a head-to-head pilot investigation. Circulation 120: 867–875. 19704093 10.1161/CIRCULATIONAHA.109.859280

[82] Gaudio C, Mirabelli F, Pelliccia F, Francone M, Tanzilli G, et al (2009) Early detection of coronary artery disease by 64-slice multidetector computed tomography in asymptomatic hypertensive high-risk patients. Int J Cardiol 135: 280–286. 18614251 10.1016/j.ijcard.2008.03.091

[83] Herzog BA, Wyss CA, Husmann L, Gaemperli O, Valenta I, et al (2009) First head-to-head comparison of effective radiation dose from low-dose 64-slice CT with prospective ECG-triggering versus invasive coronary angiography. Heart 95: 1656–1661. 19581273 10.1136/hrt.2008.162420

[84] Leschka S, Stolzmann P, Desbiolles L, Baumueller S, Goetti R, et al (2009) Diagnostic accuracy of high-pitch dual-source CT for the assessment of coronary stenoses: first experience. Eur Radiol 19: 2896–2903. 19760229 10.1007/s00330-009-1618-9

[85] Meng L, Cui L, Cheng Y, Wu X, Tang Y, et al (2009) Effect of heart rate and coronary calcification on the diagnostic accuracy of the dual-source CT coronary angiography in patients with suspected coronary artery disease. Korean J Radiol 10: 347–354. 19568462 10.3348/kjr.2009.10.4.347 PMC2702043

[86] Pontone G, Andreini D, Bartorelli AL, Cortinovis S, Mushtaq S, et al (2009) Diagnostic accuracy of coronary computed tomography angiography: a comparison between prospective and retrospective electrocardiogram triggering. Journal of the American College of Cardiology 54: 346–355. 19608033 10.1016/j.jacc.2009.04.027

[87] Reimann AJ, Tsiflikas I, Brodoefel H, Scheuering M, Rinck D, et al (2009) Efficacy of computer aided analysis in detection of significant coronary artery stenosis in cardiac using dual source computed tomography. Int J Cardiovasc Imaging 25: 195–203. 18821077 10.1007/s10554-008-9372-7

[88] Rixe J, Rolf A, Conradi G, Moellmann H, Nef H, et al (2009) Detection of relevant coronary artery disease using dual-source computed tomography in a high probability patient series: comparison with invasive angiography. Circ J 73: 316–322. 19122307 10.1253/circj.cj-08-0534

[89] Sheikh M, Ben-Nakhi A, Shukkur AM, Sinan T, Al-Rashdan I (2009) Accuracy of 64-multidetector-row computed tomography in the diagnosis of coronary artery disease. Med Princ Pract 18: 323–328. 19494542 10.1159/000215732

[90] Andreini D, Pontone G, Bartorelli AL, Agostoni P, Mushtaq S, et al (2010) Comparison of the diagnostic performance of 64-slice computed tomography coronary angiography in diabetic and non-diabetic patients with suspected coronary artery disease. Cardiovasc Diabetol 9: 80. 21114858 10.1186/1475-2840-9-80 PMC3006364

[91] Cademartiri F, Maffei E, Palumbo A, Seitun S, Martini C, et al (2010) Coronary calcium score and computed tomography coronary angiography in high-risk asymptomatic subjects: assessment of diagnostic accuracy and prevalence of non-obstructive coronary artery disease. Eur Radiol 20: 846–854. 19760230 10.1007/s00330-009-1612-2

[92] Cademartiri F, Maffei E, Palumbo A, Martini C, Seitun S, et al (2010) Diagnostic accuracy of computed tomography coronary angiography in patients with a zero calcium score. European radiology 20: 81–87. 19657651 10.1007/s00330-009-1529-9

[93] Carrascosa P, Capuñay C, Deviggiano A, Goldsmit A, Tajer C, et al (2010) Accuracy of low-dose prospectively gated axial coronary CT angiography for the assessment of coronary artery stenosis in patients with stable heart rate. Journal of Cardiovascular Computed Tomography 4: 197–205. 20444666 10.1016/j.jcct.2010.04.001

[94] Chen HW, Fang XM, Hu XY, Bao J, Hu CH, et al (2010) Efficacy of dual-source CT coronary angiography in evaluating coronary stenosis: initial experience. Clin Imaging 34: 165–171. 20416479 10.1016/j.clinimag.2009.05.012

[95] de Graaf FR, Schuijf JD, van Velzen JE, Kroft LJ, de Roos A, et al (2010) Diagnostic accuracy of 320-row multidetector computed tomography coronary angiography in the non-invasive evaluation of significant coronary artery disease. Eur Heart J 31: 1908–1915. 20047991 10.1093/eurheartj/ehp571

[96] Donati OF, Scheffel H, Stolzmann P, Baumuller S, Plass A, et al (2010) Combined cardiac CT and MRI for the comprehensive workup of hemodynamically relevant coronary stenoses. AJR Am J Roentgenol 194: 920–926. 20308492 10.2214/AJR.09.3225

[97] Fang XM, Chen HW, Hu XY, Bao J, Chen Y, et al (2010) Dual-source CT coronary angiography without heart rate or rhythm control in comparison with conventional coronary angiography. Int J Cardiovasc Imaging 26: 323–331. 10.1007/s10554-009-9527-1 19888670

[98] Husmann L, Herzog BA, Burger IA, Buechel RR, Pazhenkottil AP, et al (2010) Usefulness of additional coronary calcium scoring in low-dose CT coronary angiography with prospective ECG-triggering: impact on total effective radiation dose and diagnostic accuracy. Academic radiology 17: 201–206. 19944630 10.1016/j.acra.2009.09.007

[99] Kajander S, Joutsiniemi E, Saraste M, Pietila M, Ukkonen H, et al (2010) Cardiac positron emission tomography/computed tomography imaging accurately detects anatomically and functionally significant coronary artery disease. Circulation 122: 603–613. 20660808 10.1161/CIRCULATIONAHA.109.915009

[100] Nasis A, Leung MC, Antonis PR, Cameron JD, Lehman SJ, et al (2010) Diagnostic accuracy of noninvasive coronary angiography with 320-detector row computed tomography. Am J Cardiol 106: 1429–1435. 21059432 10.1016/j.amjcard.2010.06.073

[101] Nazeri I, Shahabi P, Tehrai M, Sharif-Kashani B, Nazeri A (2010) Impact of calcification on diagnostic accuracy of 64-slice spiral computed tomography for detecting coronary artery disease: a single center experience. Arch Iran Med 13: 373–383. 20804303

[102] Ovrehus KA, Munkholm H, Bottcher M, Botker HE, Norgaard BL (2010) Coronary computed tomographic angiography in patients suspected of coronary artery disease: impact of observer experience on diagnostic performance and interobserver reproducibility. J Cardiovasc Comput Tomogr 4: 186–194. 20451487 10.1016/j.jcct.2010.03.010

[103] Sato A, Nozato T, Hikita H, Miyazaki S, Takahashi Y, et al (2010) Incremental value of combining 64-slice computed tomography angiography with stress nuclear myocardial perfusion imaging to improve noninvasive detection of coronary artery disease. J Nucl Cardiol 17: 19–26. 19777317 10.1007/s12350-009-9150-5

[104] Scheffel H, Stolzmann P, Plass A, Leschka S, Grunenfelder J, et al (2010) Coronary artery disease in patients with cardiac tumors: preoperative assessment by computed tomography coronary angiography. Interact Cardiovasc Thorac Surg 10: 513–518. 20118120 10.1510/icvts.2009.227439

[105] Scheffel H, Stolzmann P, Alkadhi H, Azemaj N, Plass A, et al (2010) Low-dose CT and cardiac MR for the diagnosis of coronary artery disease: accuracy of single and combined approaches. Int J Cardiovasc Imaging 26: 579–590. 20146002 10.1007/s10554-010-9595-2

[106] Xu Y, Tang L, Zhu X, Xu H, Tang J, et al (2010) Comparison of dual-source CT coronary angiography and conventional coronary angiography for detecting coronary artery disease. Int J Cardiovasc Imaging 26 Suppl 175–81. 20049537 10.1007/s10554-009-9568-5

[107] Yang X, Gai LY, Li P, Chen YD, Li T, et al (2010) Diagnostic accuracy of dual-source CT angiography and coronary risk stratification. Vasc Health Risk Manag 6: 935–941. 21057578 10.2147/VHRM.S13879 PMC2964946

[108] Zhang LJ, Wu SY, Wang J, Lu Y, Zhang ZL, et al (2010) Diagnostic accuracy of dual-source CT coronary angiography: The effect of average heart rate, heart rate variability, and calcium score in a clinical perspective. Acta Radiol 51: 727–740. 20707657 10.3109/02841851.2010.492792

[109] Achenbach S, Goroll T, Seltmann M, Pflederer T, Anders K, et al (2011) Detection of coronary artery stenoses by low-dose, prospectively ECG-triggered, high-pitch spiral coronary CT angiography. JACC Cardiovasc Imaging 4: 328–337. 21492807 10.1016/j.jcmg.2011.01.012

[110] Bamberg F, Becker A, Schwarz F, Marcus RP, Greif M, et al (2011) Detection of Hemodynamically Significant Coronary Artery Stenosis: Incremental Diagnostic Value of Dynamic CT-based Myocardial Perfusion Imaging. Radiology 260: 689–698. 21846761 10.1148/radiol.11110638

[111] Gang S, Min L, Li L, Guo-Ying L, Lin X, et al. (2011) Evaluation of CT coronary artery angiography with 320-row detector CT in a high-risk population. Br J Radiol. 10.1259/bjr/90347290 PMC3479877 21304010

[112] Kerl JM, Schoepf UJ, Zwerner PL, Bauer RW, Abro JA, et al (2011) Accuracy of coronary artery stenosis detection with CT versus conventional coronary angiography compared with composite findings from both tests as an enhanced reference standard. European radiology 21: 1895–1903. 21533864 10.1007/s00330-011-2134-2

[113] Moon JH, Park E-A, Lee W, Yin YH, Chung JW, et al (2011) The Diagnostic Accuracy, Image Quality and Radiation Dose of 64-Slice Dual-Source CT in Daily Practice: a Single Institution's Experience. Korean Journal of Radiology 12: 308–318. 21603290 10.3348/kjr.2011.12.3.308 PMC3088848

[114] Stolzmann P, Goetti R, Baumueller S, Plass A, Falk V, et al (2011) Prospective and retrospective ECG-gating for CT coronary angiography perform similarly accurate at low heart rates. Eur J Radiol 79: 85–91. 20079993 10.1016/j.ejrad.2009.12.016

[115] van Velzen JE, Schuijf JD, de Graaf FR, Boersma E, Pundziute G, et al (2011) Diagnostic performance of non-invasive multidetector computed tomography coronary angiography to detect coronary artery disease using different endpoints: detection of significant stenosis vs. detection of atherosclerosis. Eur Heart J 32: 637–645. 21037254 10.1093/eurheartj/ehq395

[116] van Velzen J, de Graaf F, Kroft L, de Roos A, Reiber JHC, et al. (2011) Performance and efficacy of 320-row computed tomography coronary angiography in patients presenting with acute chest pain: results from a clinical registry. The International Journal of Cardiovascular Imaging (formerly Cardiac Imaging): 1–12. 10.1007/s10554-011-9889-z PMC3360867 21614485

[117] Vavere AL, Arbab-Zadeh A, Rochitte CE, Dewey M, Niinuma H, et al (2011) Coronary Artery Stenoses: Accuracy of 64–Detector Row CT Angiography in Segments with Mild, Moderate, or Severe Calcification—A Subanalysis of the CORE-64 Trial. Radiology 261: 100–108. 21828192 10.1148/radiol.11110537 PMC3176425

[118] Xu L, Yang L, Fan Z, Yu W, Lv B, et al (2011) Diagnostic performance of 320-detector CT coronary angiography in patients with atrial fibrillation: preliminary results. Eur Radiol 21: 936–943. 21153826 10.1007/s00330-010-1987-0

[119] Zhang T, Luo Z, Wang D, Han D, Bai J, et al (2011) Radiation dose in coronary artery angiography with 320-detector row CT and its diagnostic accuracy: comparison with 64-detector row CT. Minerva Med 102: 249–259. 21959699

[120] Dharampal AS, Papadopoulou SL, Rossi A, Weustink AC, Mollet NR, et al (2012) Computed tomography coronary angiography accuracy in women and men at low to intermediate risk of coronary artery disease. Eur Radiol 22: 2415–2423. 22669338 10.1007/s00330-012-2503-5 PMC3472076

[121] Kadokami T, Ando S, Momii H, Yoshida M, Narita S, et al (2012) Diagnostic performance of cardiac fusion images from myocardial perfusion imaging and multislice computed tomography coronary angiography for assessment of hemodynamically significant coronary artery lesions: an observational study. Nucl Med Commun 33: 60–68. 22008633 10.1097/MNM.0b013e32834d3bde

[122] Kerl JM, Schoepf UJ, Bauer RW, Tekin T, Costello P, et al (2012) 64-slice multidetector-row computed tomography in the diagnosis of coronary artery disease: interobserver agreement among radiologists with varied levels of experience on a per-patient and per-segment basis. J Thorac Imaging 27: 29–35. 21102356 10.1097/RTI.0b013e3181f82805

[123] Maffei E, Martini C, Rossi A, Mollet N, Lario C, et al (2012) Diagnostic accuracy of second-generation dual-source computed tomography coronary angiography with iterative reconstructions: a real-world experience. Radiol Med 117: 725–738. 22095423 10.1007/s11547-011-0754-x

[124] Maffei E, Martini C, Tedeschi C, Spagnolo P, Zuccarelli A, et al (2012) Diagnostic accuracy of 64-slice computed tomography coronary angiography in a large population of patients without revascularisation: registry data on the comparison between male and female population. Radiol Med 117: 6–18. 21643636 10.1007/s11547-011-0693-6

[125] Sohns C, Kruse S, Vollmann D, Luthje L, Dorenkamp M, et al (2012) Accuracy of 64-multidetector computed tomography coronary angiography in patients with symptomatic atrial fibrillation prior to pulmonary vein isolation. Eur Heart J Cardiovasc Imaging 13: 263–270. 22146783 10.1093/ejechocard/jer277

[126] Uehara M, Takaoka H, Kobayashi Y, Funabashi N (2012) Diagnostic accuracy of 320-slice computed-tomography for detection of significant coronary artery stenosis in patients with various heart rates and heart rhythms compared with conventional coronary-angiography. Int J Cardiol. 10.1016/j.ijcard.2012.02.017 22429616

[127] van Velzen JE, de Graaf FR, Kroft LJ, de Roos A, Reiber JH, et al (2012) Performance and efficacy of 320-row computed tomography coronary angiography in patients presenting with acute chest pain: results from a clinical registry. Int J Cardiovasc Imaging 28: 865–876. 21614485 10.1007/s10554-011-9889-z PMC3360867

[128] Gueret P, Deux JF, Bonello L, Sarran A, Tron C, et al (2013) Diagnostic performance of computed tomography coronary angiography (from the Prospective National Multicenter Multivendor EVASCAN Study). Am J Cardiol 111: 471–478. 23261002 10.1016/j.amjcard.2012.10.029

[129] Pelliccia F, Pasceri V, Evangelista A, Pergolini A, Barilla F, et al (2013) Diagnostic accuracy of 320-row computed tomography as compared with invasive coronary angiography in unselected, consecutive patients with suspected coronary artery disease. Int J Cardiovasc Imaging 29: 443–452. 22806317 10.1007/s10554-012-0095-4

