# Supplementary figures and images for: The Diagnostic Performance of Coronary Artery Angiography with 64-MSCT and Post 64-MSCT: Systematic Review and Meta-Analysis
Source: PLoS One. 2014 Jan 21;9(1):e84937. doi: 10.1371/journal.pone.0084937 (PMC3897406; doi:10.1371/journal.pone.0084937)

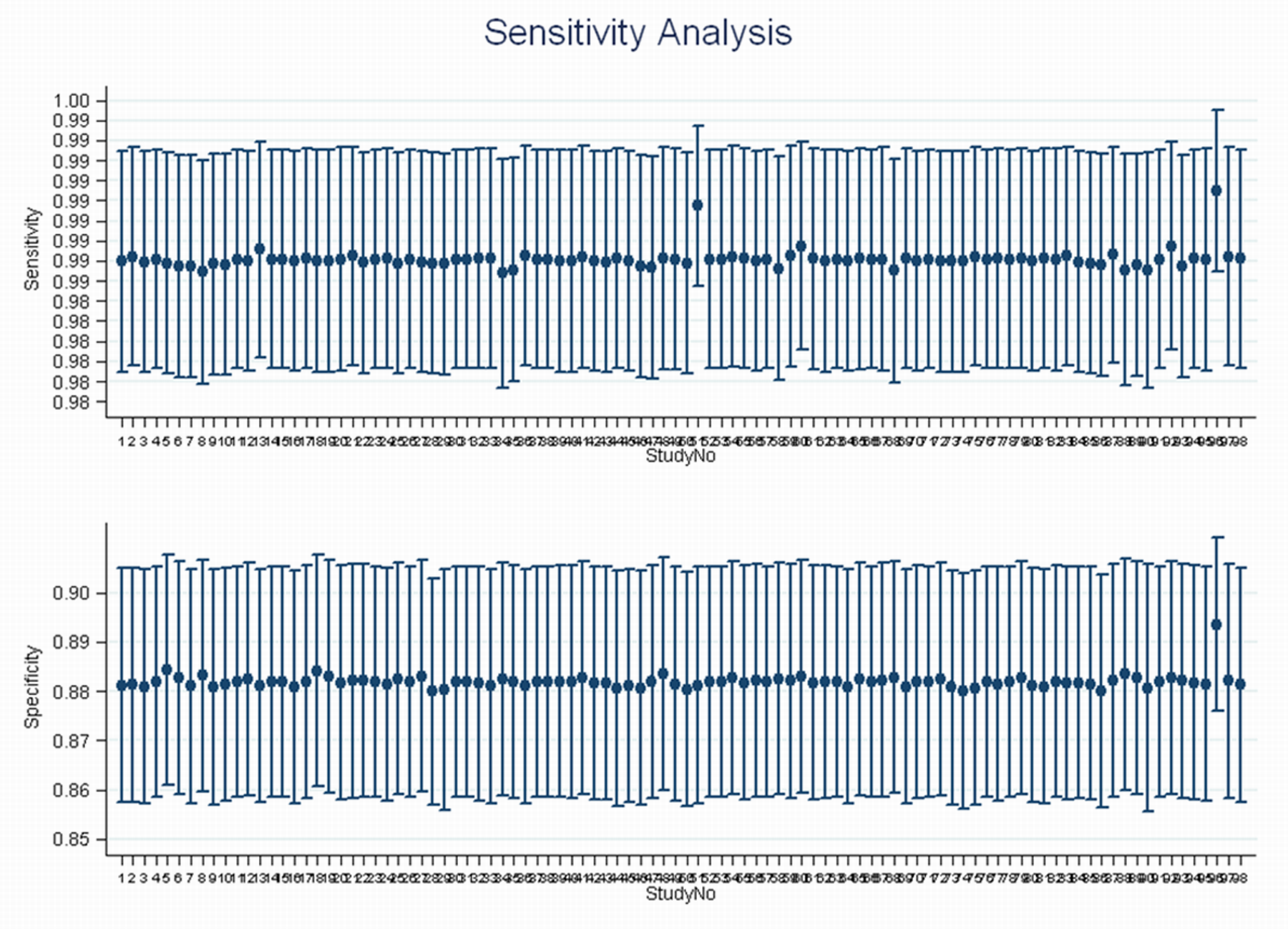

Supplement: Figure S1 — The Serrbar Illustrating a Sensitivity Analysis in which the Meta-Analysis was Re-estimated by Omitting Each Study in Turn. The sensitivity analysis indicates that no study influenced the pooled sensitivity and specificity larger than 0.02. (TIF) [file pone.0084937.s001.tif]
