# Supplementary material for: The Diagnostic Performance of Coronary Artery Angiography with 64-MSCT and Post 64-MSCT: Systematic Review and Meta-Analysis
Source: PLoS One. 2014 Jan 21;9(1):e84937. doi: 10.1371/journal.pone.0084937 (PMC3897406; doi:10.1371/journal.pone.0084937)
Supplement: Table S1 — Quality Assessment of Studies Enrolled to Diagnostic Accuracy. (DOCX) [file pone.0084937.s002.docx]

Table S1. Quality Assessment of Studies Enrolled to Diagnostic Accuracy

| **Authors** | **Item1** | **Item2** | **Item3** | **Item4** | **Item5** | **Item6** | **Item7** | **Item8** | **Item9** | **Item10** | **Item11** | **Item12** | **Item13** | **Item14** |
| --- | --- | --- | --- | --- | --- | --- | --- | --- | --- | --- | --- | --- | --- | --- |
| Leber[[1](#_ENREF_1)] | Y | Y | Y | Y | Y | Y | Y | Y | Y | U | Y | U | Y | N |
| Leschka[[2](#_ENREF_2)] | Y | Y | Y | Y | Y | Y | Y | Y | Y | Y | Y | N | Y | N |
| Mollet[[3](#_ENREF_3)] | Y | Y | Y | Y | Y | Y | Y | N | Y | Y | Y | U | Y | Y |
| Pugliese[[4](#_ENREF_4)] | Y | Y | Y | Y | Y | Y | Y | Y | N | Y | Y | U | N | N |
| Raff[[5](#_ENREF_5)] | Y | N | Y | Y | Y | Y | Y | N | N | Y | Y | N | N | N |
| Ehara[[6](#_ENREF_6)] | Y | Y | Y | Y | Y | Y | Y | Y | N | Y | Y | U | Y | N |
| Ghostine[[7](#_ENREF_7)] | U | Y | Y | Y | Y | Y | Y | Y | Y | Y | Y | U | U | N |
| Meijboom[[8](#_ENREF_8)] | U | Y | Y | U | Y | Y | Y | Y | N | Y | U | N | U | N |
| Nikolaou[[9](#_ENREF_9)] | Y | Y | Y | U | Y | Y | Y | Y | N | Y | Y | N | N | Y |
| Plass[[10](#_ENREF_10)] | N | Y | Y | U | Y | Y | Y | Y | Y | Y | Y | N | N | N |
| Ropers[[11](#_ENREF_11)] | N | N | Y | Y | Y | Y | Y | N | N | U | U | U | N | N |
| Scheffel[[12](#_ENREF_12)] | Y | Y | Y | Y | Y | Y | Y | Y | N | U | Y | U | N | N |
| Schuijf[[13](#_ENREF_13)] | Y | Y | Y | Y | Y | Y | Y | Y | N | Y | Y | U | N | N |
| Cademartiri[[14](#_ENREF_14)] | N | Y | Y | Y | Y | Y | Y | Y | N | Y | U | U | Y | N |
| Herzog[[15](#_ENREF_15)] | Y | Y | Y | U | Y | Y | Y | N | N | Y | Y | N | U | Y |
| Herzog[[16](#_ENREF_16)] | Y | Y | Y | N | Y | Y | Y | Y | U | Y | Y | N | N | Y |
| Heuschmid[[17](#_ENREF_17)] | N | N | Y | Y | Y | Y | Y | Y | N | U | U | U | N | N |
| Johnson[[18](#_ENREF_18)] | Y | Y | Y | Y | Y | Y | Y | Y | U | Y | U | U | N | N |
| Leber[[19](#_ENREF_19)] | U | Y | Y | Y | Y | Y | Y | N | N | U | U | U | N | N |
| Meijboom1[[20](#_ENREF_20)] | Y | Y | Y | Y | Y | Y | Y | Y | N | U | U | U | N | Y |
| Meijboom2[[21](#_ENREF_21)] | U | Y | Y | Y | Y | Y | Y | Y | N | Y | Y | U | N | Y |
| Oncel1[[22](#_ENREF_22)] | Y | Y | Y | Y | Y | Y | Y | U | Y | Y | Y | U | Y | Y |
| Ropers[[23](#_ENREF_23)] | Y | Y | Y | Y | Y | Y | Y | Y | N | Y | U | N | Y | N |
| Scheffel[[24](#_ENREF_24)] | U | Y | Y | Y | Y | Y | Y | Y | N | Y | Y | U | N | Y |
| Schlosser[[25](#_ENREF_25)] | U | Y | Y | Y | Y | Y | Y | Y | N | Y | U | N | N | Y |
| Weustink[[26](#_ENREF_26)] | N | Y | Y | Y | Y | Y | Y | Y | N | Y | U | U | Y | Y |
| Achenbach1[[27](#_ENREF_27)] | Y | Y | Y | U | Y | Y | Y | N | N | Y | U | U | N | N |
| Achenbach2[[27](#_ENREF_27)] | Y | Y | Y | U | Y | Y | Y | Y | N | Y | U | U | N | N |
| Alkadhi[[28](#_ENREF_28)] | Y | Y | Y | Y | Y | Y | Y | Y | N | U | Y | N | Y | N |
| Brodoefel[[29](#_ENREF_29)] | Y | Y | Y | Y | Y | Y | Y | N | Y | Y | Y | N | N | Y |
| Han[[30](#_ENREF_30)] | Y | Y | Y | U | N | Y | Y | Y | Y | U | Y | U | U | Y |
| Herzog[[31](#_ENREF_31)] | Y | Y | Y | Y | Y | Y | Y | Y | Y | U | Y | U | Y | Y |
| Leschka1[[32](#_ENREF_32)] | Y | Y | Y | N | Y | Y | Y | N | N | Y | Y | U | Y | Y |
| Leschka2[[33](#_ENREF_33)] | Y | Y | Y | Y | Y | Y | Y | Y | N | Y | Y | N | Y | Y |
| Maruyama1[[34](#_ENREF_34)] | Y | Y | Y | U | Y | Y | Y | Y | N | U | U | U | N | Y |
| Maruyama2[[34](#_ENREF_34)] | Y | Y | Y | U | Y | Y | Y | Y | N | U | U | U | N | Y |
| Miller[[35](#_ENREF_35)] | Y | Y | Y | Y | Y | Y | Y | U | Y | Y | Y | U | Y | Y |
| Pugliese[[36](#_ENREF_36)] | Y | Y | Y | Y | Y | Y | Y | N | Y | Y | U | U | Y | Y |
| Pundziute[[37](#_ENREF_37)] | Y | Y | Y | U | Y | Y | Y | Y | N | Y | Y | U | U | N |
| Ravipati[[38](#_ENREF_38)] | U | N | Y | U | Y | Y | Y | U | U | U | U | U | Y | N |
| Scheffel[[39](#_ENREF_39)] | Y | Y | Y | Y | Y | Y | Y | Y | U | Y | Y | U | N | Y |
| Stolzmann[[40](#_ENREF_40)] | Y | Y | Y | Y | Y | Y | Y | Y | N | Y | Y | N | N | Y |
| Dewey[[41](#_ENREF_41)] | Y | Y | Y | Y | Y | Y | Y | Y | Y | Y | Y | U | Y | U |
| Gaudio[[42](#_ENREF_42)] | U | U | Y | Y | Y | Y | Y | U | U | U | Y | U | Y | Y |
| Herzog[[43](#_ENREF_43)] | Y | Y | Y | Y | Y | Y | Y | Y | Y | U | Y | U | Y | Y |
| Leschka[[44](#_ENREF_44)] | Y | Y | Y | Y | Y | Y | Y | Y | Y | Y | Y | N | Y | Y |
| Meng[[45](#_ENREF_45)] | Y | Y | Y | Y | Y | Y | Y | U | Y | Y | U | N | Y | N |
| Pontone1[[46](#_ENREF_46)] | Y | Y | Y | Y | Y | Y | Y | N | N | Y | Y | U | Y | Y |
| Pontone2[[46](#_ENREF_46)] | Y | Y | Y | Y | Y | Y | Y | Y | N | Y | Y | U | Y | Y |
| Reimann[[47](#_ENREF_47)] | U | U | Y | Y | Y | Y | Y | Y | N | U | U | U | N | U |
| Rixe[[48](#_ENREF_48)] | U | Y | Y | U | Y | Y | Y | N | Y | Y | N | N | N | Y |
| Sheikh[[49](#_ENREF_49)] | Y | N | Y | Y | Y | Y | Y | N | N | U | Y | U | Y | N |
| Weustink1[[50](#_ENREF_50)] | Y | Y | Y | U | Y | Y | Y | N | U | Y | Y | U | Y | U |
| Weustink2[[50](#_ENREF_50)] | Y | Y | Y | U | Y | Y | Y | Y | U | Y | Y | U | Y | U |
| Alkadhi1[[51](#_ENREF_51)] | Y | Y | Y | Y | Y | Y | Y | Y | N | Y | Y | N | Y | Y |
| Alkadhi2[[51](#_ENREF_51)] | Y | Y | Y | Y | Y | Y | Y | Y | N | Y | Y | N | Y | Y |
| Andreini1[[52](#_ENREF_52)] | U | Y | Y | Y | Y | Y | Y | Y | N | Y | Y | U | Y | Y |
| Andreini2[[52](#_ENREF_52)] | U | Y | Y | Y | Y | Y | Y | N | N | Y | Y | U | Y | Y |
| Cademartiri1[[53](#_ENREF_53)] | U | Y | Y | N | Y | Y | Y | Y | Y | Y | Y | U | U | N |
| Cademartiri2[[54](#_ENREF_54)] | U | N | Y | U | Y | Y | Y | U | N | Y | Y | U | U | Y |
| Carrascosa[[55](#_ENREF_55)] | Y | Y | Y | Y | Y | Y | Y | Y | Y | U | U | U | Y | Y |
| Chen[[56](#_ENREF_56)] | Y | Y | Y | Y | Y | Y | Y | U | Y | Y | Y | U | N | Y |
| de Graaf[[57](#_ENREF_57)] | Y | Y | Y | Y | Y | Y | Y | Y | Y | Y | Y | U | N | Y |
| Donati[[58](#_ENREF_58)] | Y | Y | Y | Y | Y | Y | Y | Y | Y | Y | Y | N | Y | Y |
| Fang[[59](#_ENREF_59)] | Y | U | Y | Y | Y | Y | Y | Y | N | Y | Y | U | N | Y |
| Husmann[[60](#_ENREF_60)] | Y | Y | Y | U | Y | Y | Y | Y | Y | U | U | U | Y | Y |
| Kajander[[61](#_ENREF_61)] | Y | Y | Y | Y | Y | Y | Y | Y | N | Y | Y | N | Y | Y |
| Nasis[[62](#_ENREF_62)] | Y | Y | Y | Y | Y | Y | Y | N | N | Y | Y | U | Y | Y |
| Nazeri[[63](#_ENREF_63)] | Y | Y | Y | Y | Y | Y | Y | Y | Y | Y | Y | N | U | Y |
| Ovrehus[[64](#_ENREF_64)] | Y | Y | Y | U | Y | Y | Y | U | Y | Y | Y | N | Y | Y |
| Sato[[65](#_ENREF_65)] | Y | Y | Y | U | Y | Y | Y | Y | Y | Y | U | Y | Y | Y |
| Scheffel1[[66](#_ENREF_66)] | N | Y | Y | U | Y | Y | Y | Y | Y | U | Y | U | Y | Y |
| Scheffel2[[67](#_ENREF_67)] | Y | U | Y | Y | Y | Y | Y | N | Y | Y | Y | N | U | Y |
| Xu[[68](#_ENREF_68)] | Y | N | Y | U | Y | Y | Y | Y | Y | U | Y | Y | U | N |
| Yang[[69](#_ENREF_69)] | Y | Y | Y | U | Y | Y | Y | Y | N | U | U | U | U | Y |
| Zhang[[70](#_ENREF_70)] | Y | Y | Y | Y | Y | Y | Y | Y | Y | U | U | U | Y | Y |
| Achenbach[[71](#_ENREF_71)] | Y | Y | Y | Y | Y | Y | Y | Y | N | Y | Y | N | Y | Y |
| Bamberg[[72](#_ENREF_72)] | Y | Y | Y | U | Y | Y | Y | Y | Y | Y | U | N | Y | Y |
| Gang[[73](#_ENREF_73)] | Y | Y | Y | U | Y | Y | Y | Y | N | U | Y | U | Y | Y |
| Kerl[[74](#_ENREF_74)] | Y | Y | Y | U | Y | Y | Y | Y | Y | U | Y | U | U | Y |
| Moon[[75](#_ENREF_75)] | Y | Y | Y | U | Y | Y | Y | U | Y | Y | Y | U | Y | Y |
| Stolzmann[[76](#_ENREF_76)] | Y | Y | Y | Y | Y | Y | Y | Y | Y | Y | Y | N | N | N |
| van Velzen1[[77](#_ENREF_77)] | Y | Y | Y | N | Y | Y | Y | U | Y | Y | Y | N | Y | Y |
| van Velzen2[[78](#_ENREF_78)] | N | N | Y | U | Y | Y | Y | Y | Y | Y | Y | U | Y | Y |
| Vavere[[79](#_ENREF_79)] | Y | Y | Y | Y | Y | Y | Y | Y | Y | Y | Y | N | U | Y |
| Xu[[80](#_ENREF_80)] | N | Y | Y | Y | Y | Y | Y | Y | Y | Y | Y | U | N | Y |
| Zhang1[[81](#_ENREF_81)] | Y | Y | Y | Y | Y | Y | Y | Y | N | U | U | U | U | N |
| Zhang2[[81](#_ENREF_81)] | Y | Y | Y | Y | Y | Y | Y | N | N | U | U | U | U | N |
| Dharampal[[82](#_ENREF_82)] | Y | Y | Y | Y | Y | Y | Y | Y | Y | Y | Y | N | Y | Y |
| Kadokami[[83](#_ENREF_83)] | Y | Y | Y | Y | Y | Y | Y | N | Y | U | U | N | U | Y |
| Kerl[[84](#_ENREF_84)] | Y | Y | Y | U | Y | Y | Y | Y | N | Y | Y | U | U | N |
| Maffei1[[85](#_ENREF_85)] | Y | Y | Y | Y | Y | Y | Y | Y | Y | U | U | N | Y | Y |
| Maffei2[[86](#_ENREF_86)] | Y | Y | Y | Y | Y | Y | Y | N | Y | U | U | N | Y | Y |
| Sohns[[87](#_ENREF_87)] | Y | Y | Y | Y | Y | Y | Y | Y | Y | Y | Y | N | U | Y |
| Uehara[[88](#_ENREF_88)] | Y | Y | Y | N | Y | Y | Y | Y | Y | Y | Y | N | Y | Y |
| van Velzen[[89](#_ENREF_89)] | Y | Y | Y | Y | Y | Y | Y | Y | Y | Y | Y | N | N | Y |
| Gueret[[90](#_ENREF_90)] | U | N | Y | Y | Y | Y | Y | N | Y | Y | Y | N | Y | Y |
| Pelliccia[[91](#_ENREF_91)] | U | Y | Y | Y | Y | Y | Y | Y | Y | Y | Y | N | Y | Y |

Note. —Items 1–14 represent questions that were part of the tool as follows: Item 1: Was the spectrum of patients representative of the patients who will receive the test in practice? Item 2: Were selection criteria clearly described? Item 3: Is the reference standard likely to correctly classify the target condition? Item 4: Is the time period between reference standard and index test short enough to be reasonably sure that the target condition did not change between the two tests? Item 5: Did the whole sample or a random selection of the sample receive verification using a reference standard of diagnosis? Item 6: Did patients receive the same reference standard regardless of the index test result? Item 7: Was the reference standard independent of the index test (ie, the index test did not form part of the reference standard)? Item 8: Was the execution of the index test described in sufficient detail to permit replication of the test? Item 9: Was the execution of the reference standard described in sufficient detail to permit its replication? Item 10: Were the index test results interpreted without knowledge of the results of the reference standard? Item 11: Were the reference standard results interpreted without knowledge of the results of the index test? Item 12: Were the same clinical data available when test results were interpreted as would be available when the test is used in practice? Item 13: Were uninterpretable/intermediate test results reported? Item 14: Were withdrawals from the study explained?

**References list of participating studies**

1. Leber AW, Knez A, von Ziegler F, Becker A, Nikolaou K, et al. (2005) Quantification of Obstructive and Nonobstructive Coronary Lesions by 64-Slice Computed Tomography: A Comparative Study With Quantitative Coronary Angiography and Intravascular Ultrasound. Journal of the American College of Cardiology 46: 147-154.

2. Leschka S, Alkadhi H, Plass A, Desbiolles L, Grunenfelder J, et al. (2005) Accuracy of MSCT coronary angiography with 64-slice technology: first experience. Eur Heart J 26: 1482-1487.

3. Mollet NR, Cademartiri F, van Mieghem CAG, Runza G, McFadden EP, et al. (2005) High-resolution spiral computed tomography coronary angiography in patients referred for diagnostic conventional coronary angiography. Circulation 112: 2318-2323.

4. Pugliese F, Mollet NRA, Runza G, Mieghem C, Meijboom WB, et al. (2005) Diagnostic accuracy of non-invasive 64-slice CT coronary angiography in patients with stable angina pectoris. European radiology 16: 575-582.

5. Raff GL, Gallagher MJ, O'Neill WW, Goldstein JA (2005) Diagnostic accuracy of noninvasive coronary angiography using 64-slice spiral computed tomography. Journal of the American College of Cardiology 46: 552-557.

6. Ehara M, Surmely JF, Kawai M, Katoh O, Matsubara T, et al. (2006) Diagnostic accuracy of 64-slice computed tomography for detecting angiographically significant coronary artery stenosis in an unselected consecutive patient population: comparison with conventional invasive angiography. Circulation journal: official journal of the Japanese Circulation Society 70: 564-571.

7. Ghostine S, Caussin C, Daoud B, Habis M, Perrier E, et al. (2006) Non-invasive detection of coronary artery disease in patients with left bundle branch block using 64-slice computed tomography. J Am Coll Cardiol 48: 1929-1934.

8. Meijboom WB, Mollet NR, Van Mieghem CA, Kluin J, Weustink AC, et al. (2006) Pre-operative computed tomography coronary angiography to detect significant coronary artery disease in patients referred for cardiac valve surgery. J Am Coll Cardiol 48: 1658-1665.

9. Nikolaou K, Knez A, Rist C, Wintersperger BJ, Leber A, et al. (2006) Accuracy of 64-MDCT in the diagnosis of ischemic heart disease. American Journal of Roentgenology 187: 111-117.

10. Plass A, Grunenfelder J, Leschka S, Alkadhi H, Eberli F, et al. (2006) Coronary artery imaging with 64-slice computed tomography from cardiac surgical perspective. European Journal of Cardio-Thoracic Surgery 30: 109-116.

11. Ropers D, Rixe J, Anders K, Küttner A, Baum U, et al. (2006) Usefulness of multidetector row spiral computed tomography with 64-× 0.6-mm collimation and 330-ms rotation for the noninvasive detection of significant coronary artery stenoses. The American journal of cardiology 97: 343-348.

12. Scheffel H, Alkadhi H, Plass A, Vachenauer R, Desbiolles L, et al. (2006) Accuracy of dual-source CT coronary angiography: First experience in a high pre-test probability population without heart rate control. Eur Radiol 16: 2739-2747.

13. Schuijf JD, Pundziute G, Jukema JW, Lamb HJ, van der Hoeven BL, et al. (2006) Diagnostic accuracy of 64-slice multislice computed tomography in the noninvasive evaluation of significant coronary artery disease. Am J Cardiol 98: 145-148.

14. Cademartiri F, Maffei E, Palumbo A, Malagò R, Alberghina F, et al. (2007) Diagnostic accuracy of 64-slice computed tomography coronary angiography in patients with low-to-intermediate risk. La Radiologia Medica 112: 969-981.

15. Herzog C, Zwerner PL, Doll JR, Nielsen CD, Nguyen SA, et al. (2007) Significant Coronary Artery Stenosis: Comparison on Per-Patient and Per-Vessel or Per-Segment Basis at 64-Section CT Angiography. Radiology 244: 112-120.

16. Herzog C, Nguyen SA, Savino G, Zwerner PL, Doll J, et al. (2007) Does Two-Segment Image Reconstruction at 64-Section CT Coronary Angiography Improve Image Quality and Diagnostic Accuracy? . Radiology 244: 121-129.

17. Heuschmid M, Burgstahler C, Reimann A, Brodoefel H, Mysal I, et al. (2007) Usefulness of noninvasive cardiac imaging using dual-source computed tomography in an unselected population with high prevalence of coronary artery disease. Am J Cardiol 100: 587-592.

18. Johnson TR, Nikolaou K, Busch S, Leber AW, Becker A, et al. (2007) Diagnostic accuracy of dual-source computed tomography in the diagnosis of coronary artery disease. Invest Radiol 42: 684-691.

19. Leber AW, Johnson T, Becker A, von Ziegler F, Tittus J, et al. (2007) Diagnostic accuracy of dual-source multi-slice CT-coronary angiography in patients with an intermediate pretest likelihood for coronary artery disease. Eur Heart J 28: 2354-2360.

20. Meijboom WB, van Mieghem CA, Mollet NR, Pugliese F, Weustink AC, et al. (2007) 64-slice computed tomography coronary angiography in patients with high, intermediate, or low pretest probability of significant coronary artery disease. J Am Coll Cardiol 50: 1469-1475.

21. Meijboom WB, Mollet NR, Van Mieghem CA, Weustink AC, Pugliese F, et al. (2007) 64-Slice CT coronary angiography in patients with non-ST elevation acute coronary syndrome. Heart 93: 1386-1392.

22. Oncel D, Oncel G, Tastan A, Tamci B (2007) Detection of significant coronary artery stenosis with 64-section MDCT angiography. European journal of radiology 62: 394-405.

23. Ropers U, Ropers D, Pflederer T, Anders K, Kuettner A, et al. (2007) Influence of heart rate on the diagnostic accuracy of dual-source computed tomography coronary angiography. J Am Coll Cardiol 50: 2393-2398.

24. Scheffel H, Leschka S, Plass A, Vachenauer R, Gaemperli O, et al. (2007) Accuracy of 64-slice computed tomography for the preoperative detection of coronary artery disease in patients with chronic aortic regurgitation. Am J Cardiol 100: 701-706.

25. Schlosser T, Mohrs OK, Magedanz A, Nowak B, Voigtlander T, et al. (2007) Noninvasive coronary angiography using 64-detector-row computed tomography in patients with a low to moderate pretest probability of significant coronary artery disease. Acta Radiol 48: 300-307.

26. Weustink AC, Meijboom WB, Mollet NR, Otsuka M, Pugliese F, et al. (2007) Reliable high-speed coronary computed tomography in symptomatic patients. Journal of the American College of Cardiology 50: 786-794.

27. Achenbach S, Ropers U, Kuettner A, Anders K, Pflederer T, et al. (2008) Randomized comparison of 64-slice single- and dual-source computed tomography coronary angiography for the detection of coronary artery disease. JACC Cardiovasc Imaging 1: 177-186.

28. Alkadhi H, Scheffel H, Desbiolles L, Gaemperli O, Stolzmann P, et al. (2008) Dual-source computed tomography coronary angiography: influence of obesity, calcium load, and heart rate on diagnostic accuracy. European Heart Journal 29: 766-776.

29. Brodoefel H, Burgstahler C, Tsiflikas I, Reimann A, Schroeder S, et al. (2008) Dual-source CT: effect of heart rate, heart rate variability, and calcification on image quality and diagnostic accuracy. Radiology 247: 346-355.

30. Han SC, Fang CC, Chen Y, Chen CL, Wang SP (2008) Coronary computed tomography angiography---a promising imaging modality in diagnosing coronary artery disease. J Chin Med Assoc 71: 241-246.

31. Herzog BA, Husmann L, Burkhard N, Gaemperli O, Valenta I, et al. (2008) Accuracy of low-dose computed tomography coronary angiography using prospective electrocardiogram-triggering: first clinical experience. European Heart Journal 29: 3037-3042.

32. Leschka S, Scheffel H, Husmann L, Gämperli O, Marincek B, et al. (2008) Effect of decrease in heart rate variability on the diagnostic accuracy of 64-MDCT coronary angiography. American Journal of Roentgenology 190: 1583-1590.

33. Leschka S, Scheffel H, Desbiolles L, Plass A, Gaemperli O, et al. (2008) Combining dual-source computed tomography coronary angiography and calcium scoring: added value for the assessment of coronary artery disease. Heart 94: 1154-1161.

34. Maruyama T, Takada M, Hasuike T, Yoshikawa A, Namimatsu E, et al. (2008) Radiation dose reduction and coronary assessability of prospective electrocardiogram-gated computed tomography coronary angiography: comparison with retrospective electrocardiogram-gated helical scan. Journal of the American College of Cardiology 52: 1450-1455.

35. Miller JM, Rochitte CE, Dewey M, Arbab-Zadeh A, Niinuma H, et al. (2008) Diagnostic performance of coronary angiography by 64-row CT. N Engl J Med 359: 2324-2336.

36. Pugliese F, Mollet NR, Hunink M, Cademartiri F, Nieman K, et al. (2008) Diagnostic Performance of Coronary CT Angiography by Using Different Generations of Multisection Scanners: Single-Center Experience. Radiology 246: 384-393.

37. Pundziute G, Schuijf JD, Jukema JW, van Werkhoven JM, Boersma E, et al. (2008) Gender influence on the diagnostic accuracy of 64-slice multislice computed tomography coronary angiography for detection of obstructive coronary artery disease. Heart 94: 48-52.

38. Ravipati G, Aronow WS, Lai H, Shao J, DeLuca AJ, et al. (2008) Comparison of sensitivity, specificity, positive predictive value, and negative predictive value of stress testing versus 64-multislice coronary computed tomography angiography in predicting obstructive coronary artery disease diagnosed by coronary angiography. Am J Cardiol 101: 774-775.

39. Scheffel H, Alkadhi H, Leschka S, Plass A, Desbiolles L, et al. (2008) Low-dose CT coronary angiography in the step-and-shoot mode: diagnostic performance. Heart 94: 1132-1137.

40. Stolzmann P, Scheffel H, Leschka S, Plass A, Baumuller S, et al. (2008) Influence of calcifications on diagnostic accuracy of coronary CT angiography using prospective ECG triggering. AJR Am J Roentgenol 191: 1684-1689.

41. Dewey M, Zimmermann E, Deissenrieder F, Laule M, Dubel HP, et al. (2009) Noninvasive coronary angiography by 320-row computed tomography with lower radiation exposure and maintained diagnostic accuracy: comparison of results with cardiac catheterization in a head-to-head pilot investigation. Circulation 120: 867-875.

42. Gaudio C, Mirabelli F, Pelliccia F, Francone M, Tanzilli G, et al. (2009) Early detection of coronary artery disease by 64-slice multidetector computed tomography in asymptomatic hypertensive high-risk patients. Int J Cardiol 135: 280-286.

43. Herzog BA, Wyss CA, Husmann L, Gaemperli O, Valenta I, et al. (2009) First head-to-head comparison of effective radiation dose from low-dose 64-slice CT with prospective ECG-triggering versus invasive coronary angiography. Heart 95: 1656-1661.

44. Leschka S, Stolzmann P, Desbiolles L, Baumueller S, Goetti R, et al. (2009) Diagnostic accuracy of high-pitch dual-source CT for the assessment of coronary stenoses: first experience. Eur Radiol 19: 2896-2903.

45. Meng L, Cui L, Cheng Y, Wu X, Tang Y, et al. (2009) Effect of heart rate and coronary calcification on the diagnostic accuracy of the dual-source CT coronary angiography in patients with suspected coronary artery disease. Korean J Radiol 10: 347-354.

46. Pontone G, Andreini D, Bartorelli AL, Cortinovis S, Mushtaq S, et al. (2009) Diagnostic accuracy of coronary computed tomography angiography: a comparison between prospective and retrospective electrocardiogram triggering. Journal of the American College of Cardiology 54: 346-355.

47. Reimann AJ, Tsiflikas I, Brodoefel H, Scheuering M, Rinck D, et al. (2009) Efficacy of computer aided analysis in detection of significant coronary artery stenosis in cardiac using dual source computed tomography. Int J Cardiovasc Imaging 25: 195-203.

48. Rixe J, Rolf A, Conradi G, Moellmann H, Nef H, et al. (2009) Detection of relevant coronary artery disease using dual-source computed tomography in a high probability patient series: comparison with invasive angiography. Circ J 73: 316-322.

49. Sheikh M, Ben-Nakhi A, Shukkur AM, Sinan T, Al-Rashdan I (2009) Accuracy of 64-multidetector-row computed tomography in the diagnosis of coronary artery disease. Med Princ Pract 18: 323-328.

50. Weustink AC, Mollet NR, Neefjes LA, van Straten M, Neoh E, et al. (2009) Preserved diagnostic performance of dual-source CT coronary angiography with reduced radiation exposure and cancer risk. Radiology 252: 53-60.

51. Alkadhi H, Stolzmann P, Desbiolles L, Baumueller S, Goetti R, et al. (2010) Low-dose, 128-slice, dual-source CT coronary angiography: accuracy and radiation dose of the high-pitch and the step-and-shoot mode. Heart 96: 933-938.

52. Andreini D, Pontone G, Bartorelli AL, Agostoni P, Mushtaq S, et al. (2010) Comparison of the diagnostic performance of 64-slice computed tomography coronary angiography in diabetic and non-diabetic patients with suspected coronary artery disease. Cardiovasc Diabetol 9: 80.

53. Cademartiri F, Maffei E, Palumbo A, Seitun S, Martini C, et al. (2010) Coronary calcium score and computed tomography coronary angiography in high-risk asymptomatic subjects: assessment of diagnostic accuracy and prevalence of non-obstructive coronary artery disease. Eur Radiol 20: 846-854.

54. Cademartiri F, Maffei E, Palumbo A, Martini C, Seitun S, et al. (2010) Diagnostic accuracy of computed tomography coronary angiography in patients with a zero calcium score. European radiology 20: 81-87.

55. Carrascosa P, Capuñay C, Deviggiano A, Goldsmit A, Tajer C, et al. (2010) Accuracy of low-dose prospectively gated axial coronary CT angiography for the assessment of coronary artery stenosis in patients with stable heart rate. Journal of Cardiovascular Computed Tomography 4: 197-205.

56. Chen HW, Fang XM, Hu XY, Bao J, Hu CH, et al. (2010) Efficacy of dual-source CT coronary angiography in evaluating coronary stenosis: initial experience. Clin Imaging 34: 165-171.

57. de Graaf FR, Schuijf JD, van Velzen JE, Kroft LJ, de Roos A, et al. (2010) Diagnostic accuracy of 320-row multidetector computed tomography coronary angiography in the non-invasive evaluation of significant coronary artery disease. Eur Heart J 31: 1908-1915.

58. Donati OF, Scheffel H, Stolzmann P, Baumuller S, Plass A, et al. (2010) Combined cardiac CT and MRI for the comprehensive workup of hemodynamically relevant coronary stenoses. AJR Am J Roentgenol 194: 920-926.

59. Fang XM, Chen HW, Hu XY, Bao J, Chen Y, et al. (2010) Dual-source CT coronary angiography without heart rate or rhythm control in comparison with conventional coronary angiography. Int J Cardiovasc Imaging 26: 323-331.

60. Husmann L, Herzog BA, Burger IA, Buechel RR, Pazhenkottil AP, et al. (2010) Usefulness of additional coronary calcium scoring in low-dose CT coronary angiography with prospective ECG-triggering: impact on total effective radiation dose and diagnostic accuracy. Academic radiology 17: 201-206.

61. Kajander S, Joutsiniemi E, Saraste M, Pietila M, Ukkonen H, et al. (2010) Cardiac positron emission tomography/computed tomography imaging accurately detects anatomically and functionally significant coronary artery disease. Circulation 122: 603-613.

62. Nasis A, Leung MC, Antonis PR, Cameron JD, Lehman SJ, et al. (2010) Diagnostic accuracy of noninvasive coronary angiography with 320-detector row computed tomography. Am J Cardiol 106: 1429-1435.

63. Nazeri I, Shahabi P, Tehrai M, Sharif-Kashani B, Nazeri A (2010) Impact of calcification on diagnostic accuracy of 64-slice spiral computed tomography for detecting coronary artery disease: a single center experience. Arch Iran Med 13: 373-383.

64. Ovrehus KA, Munkholm H, Bottcher M, Botker HE, Norgaard BL (2010) Coronary computed tomographic angiography in patients suspected of coronary artery disease: impact of observer experience on diagnostic performance and interobserver reproducibility. J Cardiovasc Comput Tomogr 4: 186-194.

65. Sato A, Nozato T, Hikita H, Miyazaki S, Takahashi Y, et al. (2010) Incremental value of combining 64-slice computed tomography angiography with stress nuclear myocardial perfusion imaging to improve noninvasive detection of coronary artery disease. J Nucl Cardiol 17: 19-26.

66. Scheffel H, Stolzmann P, Plass A, Leschka S, Grunenfelder J, et al. (2010) Coronary artery disease in patients with cardiac tumors: preoperative assessment by computed tomography coronary angiography. Interact Cardiovasc Thorac Surg 10: 513-518.

67. Scheffel H, Stolzmann P, Alkadhi H, Azemaj N, Plass A, et al. (2010) Low-dose CT and cardiac MR for the diagnosis of coronary artery disease: accuracy of single and combined approaches. Int J Cardiovasc Imaging 26: 579-590.

68. Xu Y, Tang L, Zhu X, Xu H, Tang J, et al. (2010) Comparison of dual-source CT coronary angiography and conventional coronary angiography for detecting coronary artery disease. Int J Cardiovasc Imaging 26 Suppl 1: 75-81.

69. Yang X, Gai LY, Li P, Chen YD, Li T, et al. (2010) Diagnostic accuracy of dual-source CT angiography and coronary risk stratification. Vasc Health Risk Manag 6: 935-941.

70. Zhang LJ, Wu SY, Wang J, Lu Y, Zhang ZL, et al. (2010) Diagnostic accuracy of dual-source CT coronary angiography: The effect of average heart rate, heart rate variability, and calcium score in a clinical perspective. Acta Radiol 51: 727-740.

71. Achenbach S, Goroll T, Seltmann M, Pflederer T, Anders K, et al. (2011) Detection of coronary artery stenoses by low-dose, prospectively ECG-triggered, high-pitch spiral coronary CT angiography. JACC Cardiovasc Imaging 4: 328-337.

72. Bamberg F, Becker A, Schwarz F, Marcus RP, Greif M, et al. (2011) Detection of Hemodynamically Significant Coronary Artery Stenosis: Incremental Diagnostic Value of Dynamic CT-based Myocardial Perfusion Imaging. Radiology 260: 689-698.

73. Gang S, Min L, Li L, Guo-Ying L, Lin X, et al. (2011) Evaluation of CT coronary artery angiography with 320-row detector CT in a high-risk population. Br J Radiol.

74. Kerl JM, Schoepf UJ, Zwerner PL, Bauer RW, Abro JA, et al. (2011) Accuracy of coronary artery stenosis detection with CT versus conventional coronary angiography compared with composite findings from both tests as an enhanced reference standard. European radiology 21: 1895-1903.

75. Moon JH, Park E-A, Lee W, Yin YH, Chung JW, et al. (2011) The Diagnostic Accuracy, Image Quality and Radiation Dose of 64-Slice Dual-Source CT in Daily Practice: a Single Institution's Experience. Korean Journal of Radiology 12: 308-318.

76. Stolzmann P, Goetti R, Baumueller S, Plass A, Falk V, et al. (2011) Prospective and retrospective ECG-gating for CT coronary angiography perform similarly accurate at low heart rates. Eur J Radiol 79: 85-91.

77. van Velzen JE, Schuijf JD, de Graaf FR, Boersma E, Pundziute G, et al. (2011) Diagnostic performance of non-invasive multidetector computed tomography coronary angiography to detect coronary artery disease using different endpoints: detection of significant stenosis vs. detection of atherosclerosis. Eur Heart J 32: 637-645.

78. van Velzen J, de Graaf F, Kroft L, de Roos A, Reiber JHC, et al. (2011) Performance and efficacy of 320-row computed tomography coronary angiography in patients presenting with acute chest pain: results from a clinical registry. The International Journal of Cardiovascular Imaging (formerly Cardiac Imaging): 1-12.

79. Vavere AL, Arbab-Zadeh A, Rochitte CE, Dewey M, Niinuma H, et al. (2011) Coronary Artery Stenoses: Accuracy of 64–Detector Row CT Angiography in Segments with Mild, Moderate, or Severe Calcification—A Subanalysis of the CORE-64 Trial. Radiology 261: 100-108.

80. Xu L, Yang L, Fan Z, Yu W, Lv B, et al. (2011) Diagnostic performance of 320-detector CT coronary angiography in patients with atrial fibrillation: preliminary results. Eur Radiol 21: 936-943.

81. Zhang T, Luo Z, Wang D, Han D, Bai J, et al. (2011) Radiation dose in coronary artery angiography with 320-detector row CT and its diagnostic accuracy: comparison with 64-detector row CT. Minerva Med 102: 249-259.

82. Dharampal AS, Papadopoulou SL, Rossi A, Weustink AC, Mollet NR, et al. (2012) Computed tomography coronary angiography accuracy in women and men at low to intermediate risk of coronary artery disease. Eur Radiol 22: 2415-2423.

83. Kadokami T, Ando S, Momii H, Yoshida M, Narita S, et al. (2012) Diagnostic performance of cardiac fusion images from myocardial perfusion imaging and multislice computed tomography coronary angiography for assessment of hemodynamically significant coronary artery lesions: an observational study. Nucl Med Commun 33: 60-68.

84. Kerl JM, Schoepf UJ, Bauer RW, Tekin T, Costello P, et al. (2012) 64-slice multidetector-row computed tomography in the diagnosis of coronary artery disease: interobserver agreement among radiologists with varied levels of experience on a per-patient and per-segment basis. J Thorac Imaging 27: 29-35.

85. Maffei E, Martini C, Rossi A, Mollet N, Lario C, et al. (2012) Diagnostic accuracy of second-generation dual-source computed tomography coronary angiography with iterative reconstructions: a real-world experience. Radiol Med 117: 725-738.

86. Maffei E, Martini C, Tedeschi C, Spagnolo P, Zuccarelli A, et al. (2012) Diagnostic accuracy of 64-slice computed tomography coronary angiography in a large population of patients without revascularisation: registry data on the comparison between male and female population. Radiol Med 117: 6-18.

87. Sohns C, Kruse S, Vollmann D, Luthje L, Dorenkamp M, et al. (2012) Accuracy of 64-multidetector computed tomography coronary angiography in patients with symptomatic atrial fibrillation prior to pulmonary vein isolation. Eur Heart J Cardiovasc Imaging 13: 263-270.

88. Uehara M, Takaoka H, Kobayashi Y, Funabashi N (2012) Diagnostic accuracy of 320-slice computed-tomography for detection of significant coronary artery stenosis in patients with various heart rates and heart rhythms compared with conventional coronary-angiography. Int J Cardiol.

89. van Velzen JE, de Graaf FR, Kroft LJ, de Roos A, Reiber JH, et al. (2012) Performance and efficacy of 320-row computed tomography coronary angiography in patients presenting with acute chest pain: results from a clinical registry. Int J Cardiovasc Imaging 28: 865-876.

90. Gueret P, Deux JF, Bonello L, Sarran A, Tron C, et al. (2013) Diagnostic performance of computed tomography coronary angiography (from the Prospective National Multicenter Multivendor EVASCAN Study). Am J Cardiol 111: 471-478.

91. Pelliccia F, Pasceri V, Evangelista A, Pergolini A, Barilla F, et al. (2013) Diagnostic accuracy of 320-row computed tomography as compared with invasive coronary angiography in unselected, consecutive patients with suspected coronary artery disease. Int J Cardiovasc Imaging 29: 443-452.
