# Supplementary material for: The Diagnostic Performance of Coronary Artery Angiography with 64-MSCT and Post 64-MSCT: Systematic Review and Meta-Analysis
Source: PLoS One. 2014 Jan 21;9(1):e84937. doi: 10.1371/journal.pone.0084937 (PMC3897406; doi:10.1371/journal.pone.0084937)
Supplement: Table S3 — Results of the Multivariate Meta-Regression Analysis for Identifying Covariates to Explain Heterogeneity at Patient Level. (DOCX) [file pone.0084937.s004.docx]

Table S3. Results of the Multivariate Meta-Regression Analysis for Identifying Covariates to Explain Heterogeneity at Patient Level

| **Parameter** | **I-squared(95%CI)** | **LRTChi** | **P value** |
| --- | --- | --- | --- |
| Gender | 84.83 (68.35 - 100.00) | 13.18 | 0.00 |
| Age | 43.57 (0.00 - 100.00) | 3.54 | 0.17 |
| 64 or post 64-MSCT | 90.47 (81.23 - 99.72) | 21.00 | 0.00 |
| Temporal resolution | 94.39 (89.67 - 99.11) | 35.64 | 0.00 |
| Heart rate | 97.19 (95.27 - 99.12) | 71.24 | 0.00 |
| Vender | 94.89 (90.71 - 99.08) | 39.16 | 0.00 |
| Scheme to evaluate the coronary arterial tree | 97.63 (96.08 - 99.17) | 84.28 | 0.00 |
| Calcium score | 99.00 (98.51 - 99.49) | 200.17 | 0.00 |
| Nonassessable segments | 98.98 (98.48 - 99.49) | 196.91 | 0.00 |
| Protocol of ECG- triggered scanning | 97.50 (95.84 - 99.16) | 79.89 | 0.00 |
| Prevalence of CAD | 63.10 (16.90 - 100.00) | 5.42 | 0.07 |

Note. —ECG=electrocardiographic, CAD=coronary artery disease
